# Understanding Endometriosis: A Broad Review of Its Causes, Management, and Impact

**DOI:** 10.3390/ijms26188878

**Published:** 2025-09-12

**Authors:** Paweł Czubak, Karolina Herda, Iwona Niewiadomska, Lechosław Putowski, Mirosław Łańcut, Maciej Masłyk

**Affiliations:** 1 Department of Molecular Biology, The John Paul II Catholic University of Lublin, ul. Konstantynów 1i, 20-708 Lublin, Poland; pawel.czubak@kul.pl; 2 Department of Biomedical and Analytical Chemistry, The John Paul II Catholic University of Lublin, ul. Konstantynów 1i, 20-708 Lublin, Poland; karolina.herda@kul.pl; 3 Institute of Psychology, The John Paul II Catholic University of Lublin, 20-950 Lublin, Poland; iwona.niewiadomska@kul.pl; 4 Chair and Department of Gynaecology and Gynaecological Endocrinology, Medical University of Lublin, Aleje Racławickie 23 (SPSW), 20-037 Lublin, Poland; putowskil@yahoo.com; 5 Experimental Research Center, The John Paul II Catholic University of Lublin, ul. Konstantynów 1i, 20-708 Lublin, Poland; miroslaw.lancut@kul.pl

**Keywords:** endometriosis, biomarkers, treatment of endometriosis, natural substances, drug targets

## Abstract

Endometriosis is a complex gynecological condition affecting 10% of women globally, characterized by the growth of endometrial-like tissue outside the uterus, leading to chronic pelvic pain, infertility, reduced quality of life, and a risk of cancer. This review examines the multifaceted aspects of endometriosis, including its causes, diagnostic approaches, and management strategies. Genetic and environmental factors, hormonal influences, and immunological dysregulation are addressed as potential drivers of disease progression. Treatment options, including pharmacological interventions (hormone therapy, pain management) and surgical interventions, are assessed for their effectiveness in alleviating symptoms and improving outcomes. Emerging therapies, such as small-molecule inhibitors and anti-angiogenic agents, are also explored. Additionally, the psychological impact of endometriosis is addressed, emphasizing the need for holistic care. This review highlights the importance of continued research to unravel the precise mechanisms underlying endometriosis and to develop targeted therapies for improved patient care.

## 1. Introduction

Endometriosis is a chronic gynecological condition where tissue similar to the lining inside the uterus, known as endometrium, starts to grow outside the uterine cavity. This ectopic endometrial tissue can be found on the ovaries, fallopian tubes, the outer surface of the uterus, and other organs within the pelvis. Rarely, it can spread beyond the pelvic region. However, endometriosis is a non-cancerous condition; it exhibits several characteristics commonly associated with malignant tumors. Like cancer, endometriosis is capable of spreading and infiltrating distant areas. It can adhere to, penetrate, and harm the tissues it affects [1]. Moreover, multiple studies have shown that women with endometriosis face a higher risk of developing epithelial ovarian cancer (EOC) [2,3,4,5,6].

This condition affects approximately 10% of women of reproductive age globally, which translates to around 176 million women worldwide. Women with a first-degree relative (mother, sister) with endometriosis are at higher risk, indicating a genetic predisposition. The exact cause of endometriosis remains uncertain, although several theories have been proposed. One widely accepted theory is retrograde menstruation, where menstrual blood flows backward through the fallopian tubes into the pelvic cavity instead of leaving the body. This allows endometrial cells to implant and grow outside the uterus. Other theories suggest that genetic, immunological, and hormonal factors play a role in the development of the disease [7,8].

The normal endometrial tissue, also known as the endometrium, is the inner lining of the uterus. It plays a critical role in the reproductive system, particularly in menstruation and pregnancy. The structure of the endometrium is complex and consists of two main layers, each with distinct cellular compositions and functions. The functional layer is the superficial layer that faces the uterine cavity and undergoes cyclic changes during the menstrual cycle. It is the layer that thickens in response to hormonal changes, supports embryo implantation, and is shed during menstruation if pregnancy does not occur. The second layer (basal layer) is the deeper layer adjacent to the myometrium (muscle layer of the uterus). Unlike the functional layer, the basal layer remains relatively constant and is not shed during menstruation. It serves as the regenerative source for the functional layer after each menstrual cycle [9].

The endometrium comprises various cell types, each playing specific roles in its function and cyclic changes [10]. Epithelial cells form the lining of the endometrial surface and glands. These cells can be ciliated, aiding in fluid movement, or secretory, producing essential secretions for early pregnancy. Stromal cells provide structural support and are involved in the hormonal response, contributing to the cyclic remodeling of the endometrium. They play a crucial role in decidualization during pregnancy. Immune cells include macrophages, natural killer cells, and T cells, which help maintain tissue homeostasis and play roles in the immune response during the menstrual cycle and pregnancy. Vascular cells, represented by endothelial cells and pericytes, form the extensive network of blood vessels that supply the endometrium, essential for nutrient delivery and tissue remodeling.

### 1.1. Theories of Endometriosis Development

**Figure 1:**
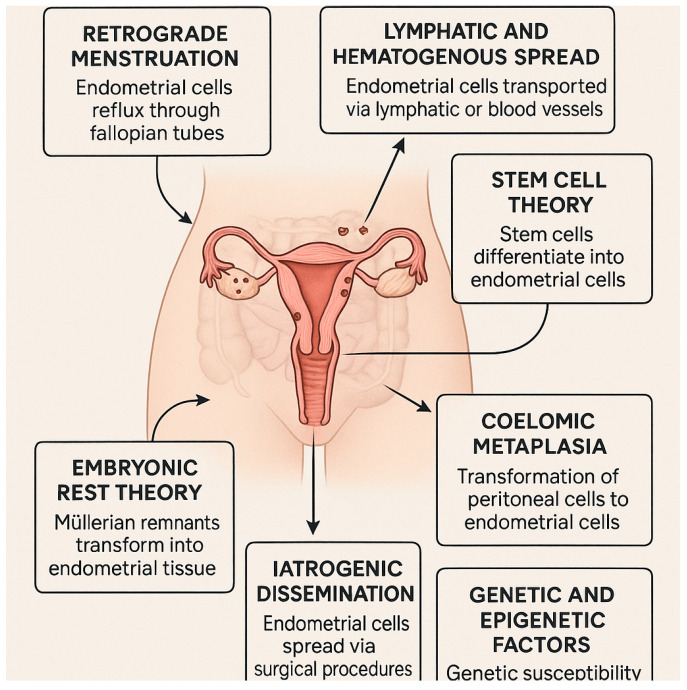
Spreading mechanisms of Endometriosis.

The exact mechanisms by which endometriosis spreads are complex and multifactorial, involving several theories and potential pathways. Here, these mechanisms and theories based on current scientific understanding are presented (Figure 1):

- Retrograde menstruation

This theory was first proposed by Dr. John Sampson in the 1920s and is one of the most widely accepted theories for the spread of endometriosis. It involves the backward flow of menstrual blood through the fallopian tubes into the pelvic cavity during menstruation. The menstrual blood contains viable endometrial cells, which implant on peritoneal surfaces and other pelvic organs, where they can proliferate and form endometriotic lesions. Because of the fact that retrograde menstruation occurs in most women, only a subset develops endometriosis, which may suggest that other factors, such as immune system dysfunction, play a role [11]. An altered immune environment, characterized by increased macrophage activity and dysregulated cytokine production, can create a permissive environment for ectopic endometrial cells to survive and thrive, preventing their elimination by the immune system. However, on the other hand, molecular and genetic data have shown that endometrial cells present in ectopic lesions and in the uterine cavity are clonally compatible, particularly with respect to mutations in the KRAS and PIK3CA genes [12,13,14]. This suggests that these cells entered the peritoneal cavity via retrograde menstruation, where they gained the ability to survive and develop. Furthermore, epigenetic changes in stromal and epithelial cells (e.g., progesterone resistance, aromatase overexpression) favor the persistence of these implants [8]. In light of this evidence, Sampson’s theory finds not only historical but also contemporary support in the form of genetic and epigenetic studies, confirming its validity.

- Lymphatic and Hematogenous Spread

This theory suggests that endometrial cells can spread through the lymphatic system or blood vessels to distant sites, including the lungs, brain, and skin. First, endometrial cells enter the lymphatic system or bloodstream. Next, they can travel to and implant in distant locations, potentially explaining cases of endometriosis found outside the pelvic region, which supports this theory [15].

- Stem cell theory

The stem cell theory provides a compelling framework for understanding the pathogenesis of endometriosis. This theory posits that stem or progenitor cells, originating from the endometrium, bone marrow, or Müllerian duct derivatives, play a pivotal role in the development and progression of endometriotic lesions. These cells are believed to translocate to ectopic sites through mechanisms such as retrograde menstruation, lymphovascular dissemination, and direct migration or invasion. Upon reaching these sites, they differentiate into the diverse cellular components observed in endometriotic lesions, including glandular, stromal, endothelial, and smooth muscle cells. This process is facilitated by mechanisms such as epithelial–mesenchymal transition (EMT), mesenchymal–epithelial transition (MET), and metaplasia [16,17,18,19,20,21].

In some endometriosis lesions, epithelial cells are monoclonal, indicating a single-cell origin, possibly from an endometrial stem or progenitor cell. In contrast, other lesions are polyclonal, suggesting contamination with polyclonal stromal cells or repeated seeding of the lesion by cells from other sources, such as bone marrow [22].

- Coelomic metaplasia

The coelomic metaplasia theory posits that pelvic endometriosis arises from metaplasia of the peritoneal mesothelium, stimulated by endometrial stroma. Endometrial stroma, a component of retrograde menstrual debris, is rich in growth factors and cytokines, which, hypothetically, are key drivers of this metaplastic process. The morphological observations support Nakamura et al.’s findings, indicating that endometriosis may develop through a progressive transformation of adjacent mesothelial cells [23].

The metaplastic theory proposes that pelvic endometriosis originates from the metaplastic transformation of peritoneal mesothelium [24]. Morphological observations support this, showing a progressive change from adjacent mesothelial cells. Furthermore, a novel in vitro model using human ovarian surface epithelium (OSE) cells demonstrates that endometriosis-like lesions can develop via metaplasia from OSE. In this model, coculturing OSE and ovarian stromal cells with 17β-estradiol resulted in lumen formation by OSE cells, surrounded by stromal cells in an epithelial–mesenchymal configuration. Glandular cells exhibited immunoreactivity for epithelial membrane antigen and cytokeratin, and electron microscopy revealed cilia, microvilli, and tight junctions.

- Embryonic rest theory

The assumptions of the embryonic rest theory are similar to those of coelomic metaplasia theory, although not limited to the mesothelium. According to this theory, endometriotic lesions originate from cells derived from the Müllerian or Wolffian duct [25]. Signore et al. noted that, after conducting analysis of female fetuses, the presence of ectopic endometrium outside the uterine cavity was found in 10 of 100 fetuses [26]. The locations where ectopic endometrial nests were found matched the locations where endometriosis occurs in women. At the same time, the theory may explain the rare occurrence of endometriosis in men.

- Iatrogenic dissemination

Iatrogenic dissemination refers to the spread of endometrial cells as a result of surgical procedures, such as cesarean sections, laparotomies, or laparoscopies, which can inadvertently transport endometrial cells to new sites. These cells can then implant and grow, leading to endometriotic lesions at surgical scar sites or other locations [27,28,29].

- Genetic and epigenetic factors

Endometriosis is influenced by both genetic and environmental factors, with genetic inheritance accounting for roughly 50% of disease susceptibility [30]. The 5–7% risk among first-degree relatives suggests a polygenic and multifactorial inheritance pattern. Epigenetic factors influencing the development of endometriosis include hypermethylation in the promoter region of tumor suppressor genes and hypomethylation in the promoter region of oncogenes, which have been associated with endometriosis. Moreover, histone modifications are often observed in endometriotic lesions. According to Bedrick et al., histone modification and methylation of genetic sequences occurred simultaneously, suggesting multifactorial epigenetic changes and dysregulation in the pathogenesis of endometriosis [31].

Genetic studies have identified nine loci associated with endometriosis, six of which (rs12700667 at 7p15.2, rs7521902 near WNT4, rs10859871 near VEZT, rs1537377 near CDKN2B-AS1, rs7739264 near ID4, and rs13394619 in GREBI) were confirmed as genome-wide significant. Two additional loci (FNI and 2p14) demonstrated borderline significance in moderate-to-severe cases [30]. A locus near the VEZT gene has been significantly linked to endometriosis in the general population, highlighting the role of specific genetic variants in the disease’s progression [32].

### 1.2. Newly Proposed Mechanisms of Pathogenesis

In addition, other mechanisms related to the pathogenesis of endometriosis have been proposed. The latest discoveries include the influence of neutrophils and related structures called NETs, the participation of chemokines, and aspects related to the presence of Pattern-recognition receptors (PRRs):

- NETs—Neutrophils play a crucial role in the pathogenesis of endometriosis. They mainly kill pathogens through phagocytosis and degradation, but they can also kill pathogenic microorganisms by releasing a network structure called NETs. NETs are different from phagocytosis and degradation and are a new way of immune response. They play a crucial role in the occurrence and development of various diseases, including endometriosis. When the integrity of endometrial epithelial cells is destroyed, it is more conducive to the invasion and infection of pathogenic microorganisms. Neutrophils can reach the uterine cavity through the endometrium and make an early response to the infection caused by the pathogens. The number of neutrophils increases in the late pregnancy of healthy dairy cows, exhibiting a strong phagocytic ability. However, when endometritis and other uterine diseases occur, the numbers and phagocytic ability of neutrophils are reduced. The formation and regulation of NETs depend on the production of ROS mediated by NADPH oxidase [33].

NETs contribute to the inflammatory microenvironment of the endometrium in endometriosis patients. They are released by neutrophils in response to mitochondrial induction and promote a more systemic effect in the disease. Additionally, the release of NET byproducts in the lesion microenvironment promotes a more inflammatory state compared to the eutopic endometrium. The increase in NETs in circulation and peritoneal fluid of endometriosis patients indicates the likelihood of a more chronic inflammatory status, which may contribute to the disease’s progression. NETs are extracellular traps formed by neutrophils in response to various stimuli. They are composed of chromatin and other proteins and are involved in the regulation of T cell function in endometriosis.

Neutrophils play a significant role in the pathogenesis of endometriosis. They are the first responders to injury and likely play a key role in early responses to lesion formation and clearance of retrograde menstruation. Neutrophils are identified as the initial responding immune cells after the induction of endometriosis, suggesting their importance in the early stages of lesion formation. Next, they release chemokines and cytokines like VEGF to promote angiogenesis by activating endothelial cells and degrading the extracellular matrix through enzymes such as elastase and cathepsin G. Moreover, they contribute to inflammation through the release of cytokines such as CXCL8/IL8 and IL6, which affect immune cell recruitment and the peritoneal cavity microenvironment [34]. Recent case-control study confirms a strong relationship between endometriosis and neutrophil extracellular traps (NETs). Endometriosis patients had significantly elevated levels of cf-DNA, nucleosomes, and neutrophil elastase (NE) in their peripheral blood compared to controls. These NET-associated markers effectively distinguished endometriosis cases and correlated with disease severity, infertility, and pain levels [35]. Moreover, neutrophils release VEGF, which promotes angiogenesis and lesion development. Other cells, like macrophages, aid in the remodeling and neo-vascularization, but the effects of neutrophils are still a factor in the early stages. Cytokines like IL6 and CXCL8/IL8 are found at abnormal levels in endometriosis and affect neutrophil recruitment and alter the peritoneal cavity microenvironment. Neutrophil autophagy, which is known to occur in human neutrophils, is also not yet linked to endometriosis [34].

- Chemokines—Chemokines play a crucial role in inflammation and immunity by stimulating the migration of immune cells, especially macrophages and granulocytes, through concentration gradients. The main sources of chemokines are activated monocytes, macrophages, and granulocytes; however, they can also be produced by many other cells of the immune system. About 50 chemokines are currently known, but the number continues to grow. The chemokines are subdivided into four main classes: the CC chemokines, the CXC chemokines, the C chemokines, and the CX3C chemokines. This classification is based on the location of the first two cysteine (C) residues and disulphide bonds in their protein sequence [36].

Chemokine/receptor systems are considered important in the pathogenesis of endometriosis. They are involved in the recruitment of immune cells into the peritoneal cavity, which is a hallmark of the disease. The constant infiltration of immune cells into the peritoneal cavity is regulated by chemokines, which direct the migration of leukocytes to the site of an ongoing inflammatory process. The role of chemokines in the development of endometriosis has been confirmed through numerous studies, and it is believed that they may also influence the incidence of endometriosis in women in the menopausal and postmenopausal periods. The concentration of selected chemokines in the peritoneal fluid of patients with endometriosis has been found to be different depending on the progressive stages of the disease, with most concentration changes being severe in women at late stages. The role of MCPs in the pathogenesis of endometriosis is still poorly understood, but some researchers have observed an increase in the concentration of this chemokine compared to that in a reference group, which they believe indicates MCP-1 participation in the development of inflammation in the peritoneal cavity [36].

In women with endometriosis, elevated levels of several chemokines—including interleukin (IL)-8, growth-related oncogene (GRO) alpha, regulated on activation, normal T expressed and secreted (RANTES), and macrophage inflammatory protein (MIP)-1—have been found in the peritoneal fluid (PF). These chemokines, specifically IL-8 and GRO alpha, along with epithelial cell-derived neutrophil-activating protein (ENA)-78, eotaxin, and interferon-inducible protein (IP)-10, may contribute to the pathogenesis of endometriosis by activating macrophages, promoting inflammatory reactions, facilitating the adhesion of endometriotic tissues within the peritoneal cavity, and enhancing angiogenesis, which is crucial for disease progression [37]. CXC chemokines play a significant role in immune surveillance in endometriosis. They are involved in the recruitment of immune cells to the site of endometriotic lesions, where they cause inflammation. CXCL1 and CXCL2 are particularly important in this process, as they are upregulated in endometriotic tissues and promote the recruitment of activated lymphocytes, neutrophils, and macrophages. CXCL1 can also cause endometrial infiltration by myeloid-derived suppressor cells (MDSCs) and neutrophils, which promote endometriotic development and angiogenesis. Additionally, CXCL12/CXCR4/CXCR7 recruit and facilitate stem cell homing from the circulation to the endometriosis, causing inflammation. CXC chemokines serve an important function in female reproductive diseases, such as PCOS and endometriosis, and their upregulation is associated with poor prognosis. Suppressing the CXCL8 and CXCL12/CXCR4/CXCR7 axis prevents the development of inflammation in both PCOS and endometriosis, which may help to improve the prognosis of disorders associated with inflammation [38].

- Pattern-recognition receptors—PRRs are a type of receptor in the innate immune system that are able to detect pathogen-associated molecular patterns (PAMPs) and danger-associated molecular patterns (DAMPs) in both intracellular and external environments. In endometriosis, PRRs play a key role in the pathological processes associated with the disease by recognizing PAMPs and triggering a receptor ligand reaction, followed by the stimulation of host immune response. This helps to remove pathogenic microorganisms from the body. There are five groups of PRRs, namely toll-like receptors (TLRs), c-type lectin receptors (CLRs), nod-like receptors (NLRs), retinoic acid-inducible gene I-like receptors (RLRs), and absent in melanoma 2 (AIM2)-like receptors (ALRs). These receptors can be divided into two major classes: membrane-bound receptors and unbound intracellular receptors. PRRs, especially TLRs, may serve as potential therapeutic targets for alleviating pain in endometriosis patients [39].

## 2. Psychological Aspects of Pain Management in Endometriosis

Studies indicate that the experience of pain accompanying endometriosis significantly correlates with psychological difficulties, the occurrence of stress, and a decrease in the quality of life of women [40]. Thus, the (subjective) psychological aspect of the occurrence of this disease is, on the one hand, a constant experience, while, on the other hand, a person-specific element due to the context of their individual, social, and cultural development [41]. The psychological manifestations of the experience of pain by women with endometriosis can be defined as a type of suffering of patients presenting for a medical diagnosis, which, however, cannot be entirely explained on the basis of physiological and/or somatic factors (e.g., experiences accompanying intense pain during menstrual bleeding, abdominal pain, sacral pain in the spine, infertility, or painful intercourse (dyspareunia)) [42].

Psychological suffering of women with endometriosis can be of a diverse nature—it can consist of: (a) anxiety and/or depression (e.g., in the form of sadness, lowered self-esteem, guilt, and helplessness) [43,44,45,46]; (b) trauma (psychological tension associated with the anticipation of pain, nightmares, and functioning in traumatic situations) [47,48]; (c) chronic stress generated by the perception of multidimensional losses (i.e., due to the high risk of difficulties in falling pregnant, loss of work, difficulties in fulfilling family/professional obligations, problems in social relations, and problems in daily activities—e.g., in the form of disturbances with sleeping, eating, movement, sexual intercourse, bowel movements, frequent fatigue, and frustration in achieving goals) [49,50]; (d) a reduction in the perceived quality of life in various domains (e.g., in the dimensions of physical functioning, mental functioning, sexual functioning, environmental functioning, social functioning, general health, vitality, fulfilled roles, and ability to work) [51,52,53,54].

According to the literature, it can be concluded that strategies combining medical and psychological interventions are more effective in reducing psychological suffering and physical pain in patients with endometriosis [55,56,57,58,59]. Psychological interventions in the care of women with endometriosis are related to taking the patients’ perspective—including areas such as: (a) respect for values and specific needs; (b) appropriate information, communication, and education; (c) providing emotional support to alleviate negative emotional states; (d) involving loved ones in care; (e) increasing the competence of medical personnel (mainly in the areas of building trust, providing support, reinforcing the changes taking place, and providing feedback) [60,61].

Improvements in the perceived quality of life by women experiencing pain from endometriosis are often a result of synergistic efforts to: (a) receive professional help—such as participation in therapy to improve well-being, understanding and accepting the disease, changing beliefs about pain, constructive coping with the disease and problems experienced (causing both trauma and chronic stress), and learning new behaviors; (b) experiencing understanding and support from family members—mainly mothers and partners; (c) participation in support groups and/or self-help groups in the form of meetings with other women with endometriosis, (d) ability to partake in self-care—including having a positive attitude toward life, engaging in relaxation activities, exercising regularly, and making sure to eat regularly and get enough sleep [62,63].

## 3. Diagnosis and Biomarkers

The primary symptoms of endometriosis include chronic pelvic pain, dysmenorrhea (painful periods), and dyspareunia (pain during intercourse). Many women also experience heavy menstrual bleeding and infertility. Additionally, endometriosis can lead to fatigue, diarrhea, constipation, bloating, and nausea, particularly during menstrual periods. The severity of symptoms is not necessarily correlated with the extent of the disease, making diagnosis challenging [64,65]. Other less obvious symptoms of the disease include chronic fatigue, brain fog, depression, dizziness, and nausea [66]. The severity of endometriosis symptoms is not necessarily correlated with the severity and extent of the disease; moreover, more than 25% of affected women are asymptomatic, making an appropriate diagnosis much more difficult [47].

Diagnosing endometriosis can be complex and often involves a combination of patient history, physical examinations, imaging tests, and, sometimes, surgical procedures. Transvaginal ultrasound and magnetic resonance imaging (MRI) can help visualize endometriotic lesions, but the gold standard for diagnosis is laparoscopy. During this minimally invasive surgical procedure, a camera is inserted into the pelvic cavity to directly observe and biopsy suspected endometriotic tissue [67].

Based on physiopathology and location, ectopic endometrial lesions, which are made up of endometrial glands and stroma, can be classified into three distinct types, differing in pathogenesis, location, and impact on the body: superficial peritoneal endometriosis (SPE), ovarian endometrioma (OMA), and deep infiltrating endometriosis (DIE) [68]. SPE is the mildest form of the disease, involving small changes on the peritoneal surface, often asymptomatic or causing mild pain. OMAs, also called chocolate cysts, develop in the ovaries as a result of repeated episodes of bleeding into the endometrial cysts, leading to their growth and potential fertility problems. In turn, DIE is the most aggressive form of endometriosis, characterized by infiltration of deep pelvic structures, such as the rectovaginal septum, intestines, or urinary bladder, which results in severe pain, organ dysfunction, and a significant deterioration in the quality of life of patients. Understanding these differences is crucial to the effective diagnosis and treatment of this complex disease.

### 3.1. The Classifications of Endometriosis

To date, no single classification system has been able to fully and effectively categorize endometriosis due to the disease’s complex and heterogeneous nature. Among the currently available systems, the revised American Society for Reproductive Medicine (rASRM) classification is the most widely used. It provides a general framework based on the number, size, and location of endometrial lesions and adhesions, classifying endometriosis into four stages: minimal, mild, moderate, and severe. While helpful for patient communication and broadly assessing disease severity, it has limitations in describing deep-infiltrating lesions and predicting surgical complexity or fertility outcomes [69].

To address these gaps, the ENZIAN classification was developed to provide a more detailed anatomical description of deep infiltrating endometriosis (DIE), especially involving retroperitoneal structures. It categorizes the disease into five stages (P1 to P5), allowing for more precise surgical planning. Importantly, the ENZIAN classification can potentially be assessed through imaging modalities such as MRI and ultrasound, making it useful preoperatively. However, further external validation studies are necessary to confirm its clinical utility [70].

Another classification tool, the Endometriosis Fertility Index (EFI), was specifically designed to predict fertility outcomes after surgically confirmed endometriosis. The EFI score ranges from 0 to 10, with higher scores indicating better fertility prognosis. It can also be useful in predicting in vitro fertilization (IVF) outcomes in affected women, thereby aiding in fertility counseling and management planning [71].

In 2021, the American Association of Gynecologic Laparoscopists (AAGL) introduced a new anatomy-based endometriosis classification system aimed at correlating anatomical findings with surgical complexity. This user-friendly scoring system incorporates a four-level scale of surgical complexity, categorizing each procedure based on its most complex surgical task. The scale was developed through extensive consultation with the AAGL Endometriosis Special Interest Group and was validated by 31 international experts. For instance, a surgery involving excision of an ovarian endometrioma, appendectomy, and ureterolysis would be considered level C [72].

To refine this system, receiver operating characteristic (ROC) curves were used to determine three cut points in the distribution of total AAGL scores, creating four defined stages. These cutoff points were selected to maximize the accuracy in distinguishing among complexity levels (A vs. B/C/D, A/B vs. C/D, and A/B/C vs. D). As a result, the AAGL 2021 classification system offers a practical and standardized way to describe surgical complexity and anatomy in endometriosis patients [72].

Together, these classification systems—rASRM, ENZIAN, EFI, and AAGL—each offer unique strengths. While none are comprehensive on their own, they contribute valuable perspectives in understanding, managing, and treating endometriosis across different clinical scenarios.

### 3.2. Protein Markers of Endometriosis

Identification of crucial protein markers in endometriosis could have a significant impact on disease treatment in several ways. In early diagnosis, they can help detect diseases at an early stage, and by understanding the unique protein expression profiles of individual patients, healthcare providers can tailor treatments to be more effective. Moreover, protein markers can provide information about the likely course of a disease. Levels of particular proteins can indicate disease severity or progression, helping clinicians to predict outcomes and plan appropriate treatment strategies. Also, the identification of protein markers is crucial in drug development. They can serve as targets for new therapies or help in the stratification of patients in clinical trials, leading to more efficient and successful drug development processes.

To date, several protein markers of endometriosis have been identified and used in diagnosis of this condition (Table 1).

CA 125

Cancer antigen 125 (CA 125) is one of the most studied protein markers in endometriosis. Elevated levels of CA 125 are often observed in women with endometriosis, particularly in those with advanced stages of the disease [73]. Despite its widespread use, CA 125 lacks specificity as it can also be elevated in other conditions like ovarian cancer, pelvic inflammatory disease, and pregnancy.

The CA 125 antigen (Mucin-16, Uniprot:Q8WXI7) is believed to form a protective, lubricating barrier against particles and infectious agents at mucosal surfaces [74]. It serves as the basis for a widely used serum assay to monitor ovarian epithelial cancer patients. However, its low sensitivity for stage I disease and lack of specificity limit its usefulness in early cancer detection. Furthermore, similarly elevated levels in some benign conditions render it unsuitable for population screening. The antigen is expressed in corneal and conjunctival epithelia but is overexpressed in ovarian carcinomas and low-malignant potential (LMP) tumors compared to normal ovarian tissue and adenomas [75].

Glycodelin

Glycodelin (Uniprot:P09466) is a major glycoprotein synthesized and secreted by the endometrium during the mid-luteal phase of the menstrual cycle and the first trimester of pregnancy [80,81]. It plays crucial roles in fertilization and exhibits immunomodulatory effects [82,83]. Four glycoforms (Glycodelin-S, -A, -F, and -C) have been identified, differing in glycosylation and biological activity. Glycodelin-A displays contraceptive and immunosuppressive properties. Glycodelin-C promotes sperm binding to the zona pellucida, while Glycodelin-F inhibits this binding and suppresses progesterone-induced acrosome reactions [84]. Glycodelin-S, present in seminal plasma, maintains sperm in an uncapacitated state [85]. While this protein plays a role in reproductive processes, it may contribute to the pathogenesis of endometriosis by affecting immune tolerance and cell proliferation.

Glycodelin-A showed sensitivity and specificity of around 80% in both serum and peritoneal fluid, suggesting its potential as a complementary biomarker alongside other independent markers [86]. Moreover, a panel of four endometriosis biomarkers (CA-125, VEGF, Annexin V, and gly-codelin/sICAM-1) showed 74–94% sensitivity and 55–75% specificity in both training and initial validation test sets [87].

HE4

Human epididymis protein 4 (HE4, Uniprot:Q14508) is a valuable clinical biomarker for detecting epithelial ovarian cancer (EOC) [88]. Although its clinical predictive power is well-established, its molecular role in EOC progression remains poorly understood. Elucidating HE4’s mechanistic functions could lead to novel targeted therapies. However, recommending HE4 as a therapeutic target is currently difficult due to limited research on its role in EOC progression. HE4 is expressed in various normal tissues, including the male reproductive system, respiratory tract, and nasopharynx, and it is highly expressed in several tumor cell lines (ovarian, colon, breast, lung, and renal). While initially believed to be exclusively expressed in the epididymis, this has since been shown not to be the case.

Human epididymis protein 4 (HE4) has been investigated as a potential marker for distinguishing endometriosis from cancers [89]. HE4’s distinct behavior compared to CA125 offers a crucial advantage in evaluating pelvic masses. While CA125 levels can be elevated in both endometriosis and ovarian cancer, HE4 typically remains normal in cases of endometriosis. Therefore, when investigating a pelvic mass, a normal HE4 level, in conjunction with other clinical and imaging findings, can be a valuable indicator that the mass is likely a benign endometrioma rather than ovarian cancer, helping to avoid unnecessary aggressive interventions [90].

IL-6

Interleukin-6 (IL-6, Uniprot:P05231) is a pleiotropic cytokine with diverse roles in immunity, tissue repair, and metabolism. IL-6 signals through membrane-bound IL-6R and IL6ST (gp130), activating “classic signaling,” or via soluble IL-6R and IL6ST, triggering “trans-signaling,” or via “cluster signaling” between cells [91]. IL-6 is crucial for acute phase responses, host defense (e.g., stimulating immune cell differentiation and germinal center formation), and bone homeostasis (via angiogenesis). It modulates metabolism (e.g., increasing lipolysis and improving insulin sensitivity) and potentially acts as a myokine. IL-6 also plays protective roles in liver injury and is essential for iron homeostasis by regulating hepcidin expression. While critical for normal physiological processes, excessive IL-6 contributes to various pathologies. Increased concentrations of IL-6 have been detected in the serum and peritoneal fluid of women with endometriosis, suggesting its involvement in the inflammatory milieu of the disease [92]. Its levels were significantly higher in women with endometriosis compared to those with idiopathic infertility or undergoing tubal surgery. Leptin levels also correlated positively with the endometriosis stage. These findings suggest a potential role for leptin in endometriosis pathogenesis, particularly in pain mechanisms. No association was found between peritoneal fluid leptin and idiopathic infertility.

VCAM-1

Vascular cell adhesion protein 1 (VCAM-1; Uniprot:P19320) is a single-pass type I transmembrane glycoprotein, predominantly expressed on endothelial cells, with increased expression on inflamed endothelium and immune cells (macrophages and dendritic cells) in both healthy and inflamed tissues [93]. Its expression is upregulated by pro-inflammatory cytokines (e.g., TNFα), reactive oxygen species (ROS), oxidized low-density lipoprotein, high glucose, toll-like receptor agonists, and shear stress. The protein is a key regulator of leukocyte adhesion to endothelium via integrin interactions [94]. During inflammation, it binds ligands on activated endothelial cells, triggering calcium channel activation and RAC1 signaling, which facilitates leukocyte transendothelial migration [95]. Additionally, it acts as a quality-control checkpoint for bone marrow entry, preventing immune cell destruction via MHC class I presentation [96]. Endometriotic tissues showed increased mRNA levels of TGF-β1 and VCAM-1. Reducing TGF-β1 levels in endometriotic cyst stromal cells significantly inhibited cell proliferation, migration, and invasion. Conversely, adding TGF-β1 stimulated these processes and markedly increased VCAM-1 protein expression. This TGF-β1-induced VCAM-1 expression was blocked by silencing Smad3. Finally, silencing VCAM-1 prevented the stimulatory effects of TGF-β1 on stromal cell proliferation, migration, and invasion [97].

VEGF

Vascular endothelial growth factor (VEGF, Uniprot: Q9UNS8) is present in normal endometrial tissue; however, its level is increased in response to increasing concentration of estrogen and progesterone. This protein supports angiogenesis of endometriosis tissue by activating the Wnt\beta-catenin axis, which helps to establish a new blood source for exfoliated human endometrium [98].

MCP-1

Monocyte chemoattractant protein-1 (MCP-1, Uniprot:Q6UZ82) is a chemokine that recruits monocytes to sites of tissue injury and infection. It is expressed in a variety of cell types, either constitutively or after induction by oxidative stress, cytokines, or growth factors. This chemokine has been shown to be upregulated and implicated in various diseases. The migration of monocytes from the bloodstream through the vascular endothelium is essential for normal immunological surveillance of tissues and for responding to inflammation. Elevated levels of MCP-1 have been found in the peritoneal fluid of women with endometriosis, indicating its role in the recruitment of immune cells and the inflammatory process [99,100].

Urocortin

Urocortin (UCN, Uniprot: P55089) is a neuropeptide present in the endometrium and ovaries, with research indicating similar serum levels in individuals with endometriosis and healthy controls. However, other studies have shown that serum urocortin levels are significantly higher in women with ovarian endometriosis compared to those with other benign ovarian cysts [101]. This diagnostic approach has a sensitivity of 88% and a specificity of 90%. Urocortin could be a potential biomarker for distinguishing ovarian endometriosis from other benign ovarian conditions.

### 3.3. Other Markers

CA 19-9

The CA 19-9 was initially considered as an oncofetal antigen. This sialylated form of Lewis Antigen is elevated in some types of cancers (e.g., gastrointestinal cancers, pancreatic cancer, and the malignant and benign ovarian tumors). Studies show that CA 19-9 can be used as a marker of endometriosis, related to its severity [102,103].

Estrogen level

Endometriosis is closely linked to steroid metabolism, with 17β-estradiol (E2) playing a key role in its growth, persistence, inflammation, and associated pain. While estradiol reaches endometriotic tissue through circulation, it is primarily produced locally within the lesions. This local accumulation of estrogen contributes to the development and progression of endometriotic lesions by activating estrogen receptors (ERs) [104]. The synthesis of E2 is upregulated in endometriotic tissue due to alterations in the activity of enzymes responsible for its biosynthesis and inactivation. Notably, endometriotic tissue can synthesize E2 de novo from cholesterol due to the high expression of two key enzymes: aromatase (CYP19A1) and steroidogenic acute regulatory protein (StAR). These enzymes are absent in normal endometrial cells.

Critical discussion on specificity of biomarkers for endometriosis

Most of these biomarkers are not disease-specific and reflect general inflammatory, hormonal, or invasive processes that are common across various gynecological and systemic diseases. Combining multiple markers—especially those reflecting different aspects of endometriosis pathology (e.g., inflammation, angiogenesis, and hormonal dependence)—may increase diagnostic accuracy. However, no single biomarker currently demonstrates sufficient specificity to serve as a standalone diagnostic tool for endometriosis. Further research into disease-specific molecular signatures or tissue-based diagnostics (e.g., microRNAs, proteomics) may offer improved specificity in the future. That is why further research in this area is so important.

### 3.4. Diagnostic Tests

BDNF

In endometriosis (EMs), Brain-Derived Neurotrophic Factor (BDNF) plays a significant role in the disease’s development and associated symptoms. It interacts with its high-affinity receptor, TrkB, to activate several signaling pathways, including MAPK, PI3K/AKT, and PLCγ. These pathways influence key processes such as abnormal cell apoptosis and autophagy, increased cell invasion and proliferation, angiogenesis, oxidative stress, and inflammation. Through these mechanisms, BDNF contributes to the progression of endometriotic lesions and the manifestation of symptoms, making it an important factor in understanding the pathogenesis of EMs and a potential target for early diagnosis and treatment [105]. In a study by Herranz-Blanco et al. [106], the performance of a diagnostic test for endometriosis based on serum BDNF (brain-derived neurotrophic factor) levels was assessed. BDNF levels were found to be significantly higher in patients with endometriosis, particularly in early stages (I–II), where BDNF was the only biomarker independently differentiating cases from controls. Combined with CA125 and patient clinical data, BDNF was incorporated into a multivariate diagnostic model, which achieved a sensitivity of 46.2% (95% CI: 25.5–66.8%) and a very high specificity of 100% (95% CI: 86.7–100%) in the validation study, making it a useful “rule-in” test to help confirm the suspicion of endometriosis. The test also detected 32% of cases of superficial endometriosis, which is often difficult to diagnose with imaging methods. Therefore, BDNF, although ineffective as a marker for excluding the disease, may be a valuable element of non-invasive diagnostics supporting the early diagnosis of endometriosis.

FUT4

FUT4 tests measure the expression levels of FUT4 mRNA in endometrial tissue. Elevated FUT4 mRNA levels are significantly associated with endometriosis and correlate with disease severity. The test, evaluated using qRT-PCR, demonstrates high accuracy (AUC = 0.90) in distinguishing women with endometriosis from controls, suggesting that FUT4 expression could serve as a specific molecular marker for the condition [107].

microRNAs

The development of non-invasive diagnostic tests for endometriosis has been a key focus of medical research for many years, with over 100 biomarkers evaluated to date. Among these, microRNAs—first described in 1993—have emerged as a promising candidate, supported by an increasing number of studies in tumors and neurodegenerative diseases.

MicroRNAs are small non-coding RNAs involved in regulating gene expression: when they bind to their target messenger RNAs, they can inhibit translation or promote degradation. Additionally, microRNAs are released into the extracellular environment via transport structures that protect them from ribonucleases, granting them remarkable stability. These circulating microRNAs are detectable in various body fluids, such as blood, urine, breast milk, tears, and saliva.

Recent evidence indicates a significant role of microRNAs in the pathophysiology of endometriosis, with a proven link between the dysregulation of specific microRNAs and the formation of endometriotic lesions [108]. A study by Bendifallah et al. assessed the usefulness of selected microRNAs as noninvasive diagnostic markers for endometriosis. Despite the large number of miRNAs analyzed and differences in expression between groups, no single miRNA achieved sufficient diagnostic parameters to be used as a clinical biomarker on its own. Only seven miRNAs showed an AUC > 0.6, and none met the criterion of ≥80% for both sensitivity and specificity. This means that even if some miRNAs show promising properties (e.g., miR-29b-1-5p or miR-3137), they do not provide adequate diagnostic accuracy as single markers. These results suggest that miRNAs in isolation have limited utility as noninvasive diagnostic tests for endometriosis, and the development of multicomponent models (miRNA panels or miRNAs + clinical data) and their validation in independent cohorts is necessary [109].

Electroviscerography

The Electroviscerography (EVG) was used as a non-invasive diagnostic method based on Gastrointestinal Myoelectrical Activity (GIMA) analysis to identify a biomarker specific to endometriosis. Signals were recorded using a water load satiety test (WLTT), which allows for the assessment of smooth muscle activity within a frequency range of 15 to 60 cpm. The data were processed using an artificial intelligence algorithm that utilized AUC (Area Under the Curve) values, patient age, and the results of standardized pain questionnaires. The obtained results demonstrated very high diagnostic accuracy—sensitivity and specificity reached 96–100%, while the AUC for the best predictive model was 0.9984, indicating near-perfect discrimination between patients with endometriosis and controls. These results confirmed the usefulness of EVG with GIMA analysis as a potentially groundbreaking, non-invasive diagnostic tool for the diagnosis of endometriosis [110].

### 3.5. Examples of Commercially Available Tests

In recent years, numerous non-invasive diagnostic tests for endometriosis have been developed, based on modern molecular biology technologies and multidimensional data analysis. One of the most advanced is Endotest (Ziwig, France), which analyzes microRNA present in saliva. In preliminary studies, it achieved sensitivity and specificity of 95–100%, with an AUC exceeding 0.90, confirming its high diagnostic value [111]. PromarkerEndo is a non-invasive blood test developed by Proteomics International (Australia), utilizing the analysis of 10 protein biomarkers in plasma to diagnose all stages of endometriosis. In a study published in Human Reproduction (2025) involving 805 samples, the test achieved exceptionally high diagnostic accuracy—an AUC of as much as 0.997 for stage IV and above 0.85 for stages I–III. The random forest model achieved sensitivity of up to 98% and specificity of up to 96%, making it a potential breakthrough in early diagnosis and reducing the need for laparoscopy (https://www.proteomics.com.au). Next test, Endosure, which has been evaluated in clinical trials, utilizes gastrointestinal bioelectrical activity analysis (GIMA) recorded via electroviscerography (EVG). According to the manufacturer, the diagnosis is 98–99% accurate (https://www.endosure.com).

## 4. Treatment of Endometriosis

Endometriosis remains a chronic gynecological condition with no definitive cure. However, a range of treatment strategies is available to manage symptoms, reduce lesion progression, and improve patients’ quality of life [64].

The choice of treatment depends on several factors, including symptom severity, disease localization and extent, age, fertility goals, and type of endometriotic lesions (superficial peritoneal disease, deep infiltrating endometriosis, or ovarian endometriomas).

### 4.1. Classical Treatment Methods

#### 4.1.1. Medical (Pharmacological) Management

The pharmacological treatment of endometriosis aims to relieve symptoms, particularly pelvic pain, and prevent disease recurrence following surgical intervention. Initial therapy typically includes non-steroidal anti-inflammatory drugs (NSAIDs) and hormonal agents (Table 2).

##### Hormonal Therapy

Combined oral contraceptives (COCs) are often the first-line hormonal treatment. These are administered in a continuous or extended-cycle regimen to suppress ovulation and menstruation. This limits cyclical bleeding within endometrial implants, thereby reducing dysmenorrhea, dyspareunia, and chronic pelvic pain [112]. Vaginal contraceptive rings and transdermal patches delivering estrogen/progestogen combinations are also effective alternatives.

While COCs offer symptom relief, their long-term efficacy in preventing disease progression is not conclusively proven. For patients who do not tolerate combined hormonal contraceptives or have contraindications, progestin-only therapies are a suitable alternative.

Progestins, such as medroxyprogesterone acetate (MPA), are administered orally or via long-acting depot formulations. They suppress endometrial growth and induce decidualization and atrophy of ectopic endometrial tissue. However, MPA is associated with side effects, including irregular breakthrough bleeding, decreased bone mineral density, increased thromboembolic risk, and an unpredictable return of ovarian function. In addition, a potential increased risk of meningioma has been reported, necessitating careful patient selection and follow-up [113].

Norethisterone acetate (NETA) is another cost-effective option with good efficacy, particularly in cases of deep-infiltrating endometriosis. Its benefits include significant pain relief, strong endometrial suppression, and acceptable tolerability. Nonetheless, common side effects include acne, seborrhea, weight gain, and a dose-dependent risk of venous thromboembolism. NETA has also been associated with mild liver toxicity, thus requiring the lowest effective dose and periodic liver function monitoring [114].

Dienogest is a selective progestin with high tolerability and long-term safety. It inhibits ovulation and reduces endometrial inflammation and angiogenesis. Comparable in efficacy to NETA for pain reduction, it is also associated with side effects such as irregular bleeding and mild osteopenia during long-term use [114]. Both progestins effectively decreased pain scores, but NETA resulted in a greater reduction in endometrioma size and a lower dropout rate, possibly due to its higher dosage. These findings indicate that NETA 5 mg/day could be a preferable alternative to dienogest for endometriosis treatment [115].

Danazol and gestrinone are older hormonal agents that induce a hypoestrogenic and hyperandrogenic state. They inhibit pituitary gonadotropin release, thereby suppressing ovarian steroidogenesis and inducing atrophy of endometriotic lesions. While effective, these agents cause significant androgenic side effects, including weight gain, acne, hirsutism, voice changes, and hepatotoxicity, limiting their use to select cases where newer options are not tolerated.

##### GnRH Agonists and Antagonists

Gonadotropin-releasing hormone (GnRH) agonists (e.g., leuprolide, goserelin, nafarelin) and GnRH antagonists (e.g., elagolix, relugolix, linzagolix) are considered second-line therapies. Both drug classes induce hypoestrogenism by suppressing ovarian estradiol production—agonists via receptor desensitization and antagonists via competitive inhibition [116]. Clinical effects include reduced menstrual bleeding, improved pain control, and decreased lesion size.

Although equally effective, they differ in onset and side-effect profiles. Common adverse effects include vasomotor symptoms, vaginal dryness, mood changes, headaches, and decreased bone mineral density. Due to these side effects, add-back therapy with low-dose estrogen and/or progestin is often recommended to mitigate bone loss and improve quality of life.

Currently, most GnRH antagonists and analogs used for medical purposes are administered via injections or nasal routes. Oral formulations are limited because GnRH and its analogs are peptide-based hormones, which are typically degraded in the gastrointestinal tract and have poor oral bioavailability. However, there has been research and development into oral GnRH antagonists. An example of such therapy is relugolix, which rapidly and sustainably suppresses testosterone levels [117].

##### Aromatase Inhibitors

Aromatase inhibitors (e.g., letrozole, anastrozole) block the enzyme aromatase, preventing the conversion of androgens to estrogens. This inhibits local estrogen production within endometriotic implants, especially in patients with persistent symptoms despite other therapies.

These agents are particularly useful in rectovaginal endometriosis, or when the disease is resistant to progestins and GnRH analogs. However, adverse effects such as vaginal dryness, insomnia, headaches, vasomotor symptoms, and osteoporosis necessitate caution. Combination therapy with progestins or GnRH analogs may be used to enhance efficacy while minimizing estrogen withdrawal effects.

##### NSAIDs and Pain Management

NSAIDs (e.g., ibuprofen, naproxen) are used primarily for symptom control. They reduce prostaglandin-mediated inflammation and pain, particularly in primary dysmenorrhea. While NSAIDs do not modify disease progression, they serve as adjuncts to hormonal therapy for acute symptom relief [112].

### 4.2. Surgical Management

Laparoscopy is the “gold standard” for the diagnosis of endometriosis [118]. Biopsies allow histological confirmation of the disease. Surgery for endometriosis is typically reserved for women who have persistent significant pain despite trials of medical treatments. For deep endometriosis, surgical excision is the method of choice. European Society of Human Reproduction and Embryology (ESHRE), European Society for Gynaecological Endoscopy (ESGE), and the World Endometriosis Society (WES) jointly developed recommendations on how to operate on endometrial cysts [119]. Surgery of endometriosis and endometrial cysts is a solution that requires a lot of attention and good training.

Deep-infiltrating endometriosis is located deeper in pelvic structures such as the intestines, bladder, and blood vessels. This type of endometriosis should be treated surgically by experienced surgeons, and preferably by interdisciplinary teams. The deep form of ovarian endometriosis is represented by endometrioma—an ovarian cyst surrounded by endometrial tissue. Bilateral endometriomas usually obliterate the Douglas cavity, which makes surgical procedure more difficult.

Pharmacological therapy to reduce the pain caused by deep-infiltrating endometriosis is an alternative to surgery, except for some conditions, such as intestinal endometriosis associated with sub-occlusive symptoms, ovarian cysts with a questionable ultrasound appearance, cysts, especially when the patient is older than 40 years of age, women trying to conceive a child, and those who refuse hormone therapy.

Prolonged suppression of ovulation dramatically reduces the risk of recurrence of lesions, especially in the case of endometrial ovarian cyst. Furthermore, lesions often re-grow over a patient’s reproductive lifespan [120].

Surgical approaches include ovarian cystectomy with cyst wall stripping, cyst drainage, ablation, and sclerotherapy. Ablation or excision of visible endometrial lesions may be done at the time of diagnostic laparoscopy. Endometrial cysts, it is endometrioma aspiration alone, are associated with a high recurrence rate and are not recommended. Cystectomy is associated with a lower recurrence rate, chance of reoperation, dysmenorrhea, and dyspareunia. The type of surgery depends on the size of the cyst, the patient’s age, the surgical stage, and the desire to get pregnant. Endometriomas over 4 cm should be surgically removed. Ovarian cystectomy for either endometriomas or non-endometriotic benign ovarian cysts exhibited a reduction in anti-Müllerian hormone levels following surgery. Surgery should be performed, avoiding blind coagulation of bleeding areas, especially during ovarian surgery. Precise spot bipolar coagulation is the key to achieving hemostasis during endometriosis surgery, which is to prevent unnecessary damage of healthy tissue [121]. Postoperative decline in anti-Mullerian hormone is the highest after cystectomy, but cystectomy is associated with lower rates of recurrence compared with ablation and sclerotherapy [122]. In order to spare ovarian tissue and reduce tissue loss, laser is suggested during surgeries. The optimal surgical technique for treating endometriosis has not been established, but the skill of the surgeon plays a role in achieving complete destruction or removal of endometriotic tissue and adhesions. Surgical treatment for endometriosis followed by medical therapy offers longer symptom relief than surgery alone (Table 3).

While surgical treatment of endometriosis is well-characterized, it is important to distinguish the management of adenomyosis, particularly in women of reproductive age seeking to preserve fertility, as the therapeutic approach differs in complexity and intent. Recent evidence highlights the role of conservative uterus-sparing techniques such as ad-enomyomectomy using either endometrial-stripping methods or the triple-flap technique. In a comparative study evaluating both procedures, both approaches resulted in significant symptom relief and reduction in uterine volume. However, the triple-flap method was associated with fewer intrauterine adhesions, a lower recurrence rate, and higher pregnancy success following surgery. These findings support the triple-flap technique as a preferable surgical option in fertility-preserving contexts, although careful patient selection and surgical expertise remain critical. Differentiating between these surgical strategies is essential when tailoring treatment plans for patients with coexisting endometriosis and adenomyosis [123].

#### Postoperative Management

Postoperative ovulation suppression therapy (e.g., with progestins or COCs) is recommended to reduce the risk of lesion recurrence. Studies suggest that combined surgery and medical therapy provide better long-term symptom control than either strategy alone.

### 4.3. Sclerotherapy and Alternative Approaches

Sclerotherapy is an investigational, minimally invasive treatment primarily for ovarian endometriomas. It involves injecting a sclerosing agent, such as ethanol, into the cyst to destroy the pseudocapsule and reduce recurrence [124,125]. The solution may be aspirated after a dwell time or left in situ. Although sclerotherapy shows promise, recurrence rates and safety profiles vary, and more evidence is needed before it can replace surgery.

In some women, particularly postmenopausal patients, endometriosis may regress spontaneously [126]. However, hormone replacement therapy in menopause may reactivate dormant lesions in some cases.

For women who have completed childbearing and continue to experience symptoms, a semi-conservative approach involving hysterectomy with ovarian preservation may be considered. This approach can reduce pain while avoiding surgical menopause.

Alternative therapy for endometriosis is also possible, including resveratrol use, which is a natural medicine derived from grapes. This compound induces apoptosis in endometrial stromal cells by suppressing survivin expression. Acupuncture, lifestyle changes, transcutaneous high-frequency electrical nerve stimulation, Chinese herbal medicine, vitamins B1 and B6, tropical heat, spinal manipulation, and behavioral interventions are all of interest to physicians treating endometriosis-related.

While these methods may offer additional symptom relief for some patients, more clinical research is needed to fully establish their efficacy and safety.

### 4.4. Natural Substances Used in the Treatment

Natural compounds are widely used in alternative medicine, including in the treatment of endometriosis, where they can produce satisfactory results [127,128]. Studies indicate that oxidative stress plays an important role in the pathophysiology of the disease and its associated pain. The antioxidant properties of natural compounds, such as polyphenols and secondary metabolites, have been shown in experimental models to alleviate inflammatory symptoms and reduce lesion growth in endometriosis. In addition, anti-inflammatory or vegan diets, due to their immunomodulatory properties, can be an effective dietary intervention for patients with the disease. Emerging evidence suggests that anti-inflammatory or plant-based diets may modulate immune responses and alleviate endometriosis-related symptoms, though clinical validation is limited. Among the natural compounds present in fruits and vegetables, they include curcumin, resveratrol, rutin, quercetin, kemferol, myricetin, cannabinoids, and capsaicin.

#### 4.4.1. Curcumin

**Figure 2:**
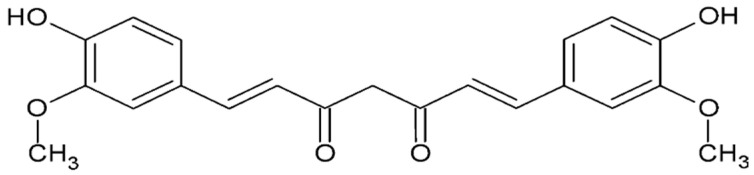
Structural formula of curcumin.

Curcumin (Figure 2), a bioactive pigment from *Curcuma longa*, has demonstrated anti-inflammatory, antioxidant, and antiproliferative effects in both in vitro and in vivo models of endometriosis [129,130,131]. These properties include the regulation of cytokine and chemokine expression, inhibition of angiogenesis, reduction of VEGF and MMP levels, and promotion of folliculogenesis.

Clinical studies using 500 mg/day of curcumin supplementation have shown reductions in pain and inflammation [132]. Although its poor bioavailability is a challenge, novel formulations may enhance its therapeutic potential. Overall, these findings suggest that curcumin may offer supportive benefits in managing endometriosis symptoms.

#### 4.4.2. Polyphenols

Polyphenols are one of the most abundant groups of natural compounds found in plants, especially fruits and vegetables. They show numerous health benefits, and their potential in the treatment of endometriosis and chronic pelvic pain (CPP) is promising due to their antioxidant and anti-inflammatory properties. In addition, their ability to affect estrogen receptors may play an important role in alleviating the symptoms of endometriosis. Studies indicate that selected polyphenols are effective in reducing pain and other discomforts associated with the disease.

- Resveratrol

**Figure 3:**
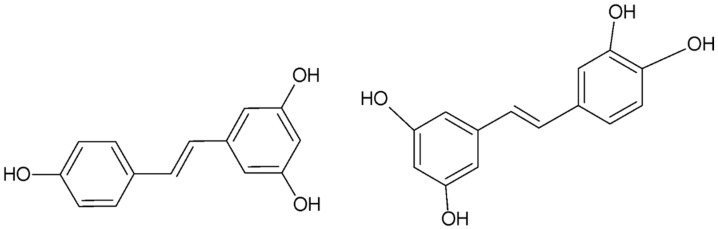
Structural formula of resveratrol.

Resveratrol (3,5,4′-trans-trihydroxystilbene, Figure 3) is a polyphenol belonging to the stilbene class, found mainly in the skin and seeds of grapes, as well as in wine, peanuts, berries, and tea. The compound is synthesized by more than 70 plant species in response to stress, injury, bacterial or fungal infections, or UV radiation, highlighting its protective role in nature [133,134]. Resveratrol in cells is converted to piceatannol, a monohydroxylated derivative of resveratrol, by the cytochrome P450 enzyme CYP1B1. In addition, cytochrome enzymes can convert resveratrol into further polyhydroxylated metabolites. Hexahydroxystilbene, which is an analog of resveratrol, exhibits as much as 6600-fold higher antiradical activity. Hydroxylation at the ortho position, as in the case of 3,4-dihydroxy-trans-stilbene, significantly enhances the ability to counteract lipoprotein peroxidation by forming hydrogen bonds between the methoxyl and phenolic groups. In mice, resveratrol decreases the number of endometrial implants when used in a dose of 6 mg/mouse [135]. Figure 3 shows the chemical formulas of resveratrol and piceatannol.

- Epigallocatechin gallate

**Figure 4:**
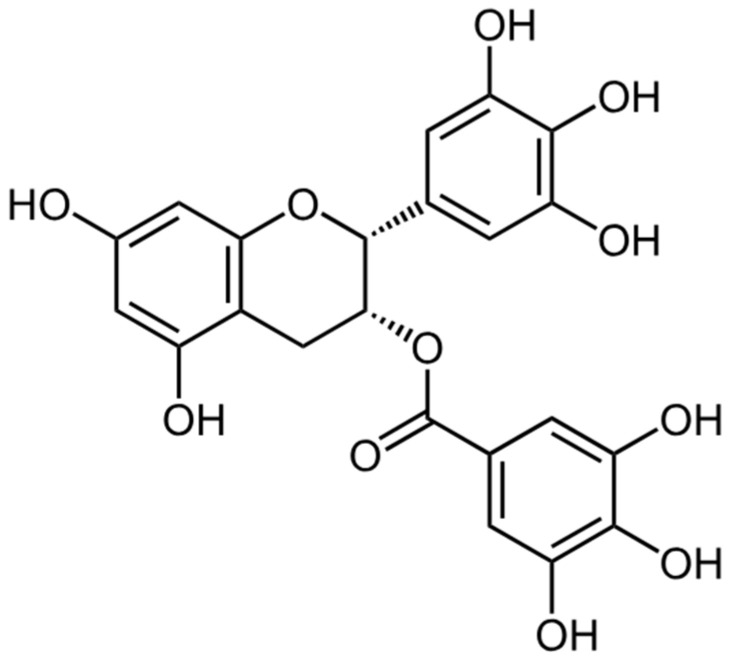
Structural formula of epigallocatechin gallate.

Epigallocatechin gallate (EGCG, Figure 4), the main flavonoid present in green tea, exhibits antioxidant, antiproliferative, and antiangiogenic properties, making it a promising compound for the treatment of endometriosis [136,137,138]. Studies have shown that EGCG reduces endometrial cell proliferation, migration, and invasion, inhibits neovascularization, and reduces fibrosis in endometriosis. In animal models, EGCG (in a dose of 50 mg/kg/day) reduced the number and volume of endometriotic lesions, although it was less effective than resveratrol. EGCG’s mechanism of action includes modulation of E-cadherin expression, inhibition of the VEGFC/VEGFR2 pathway, and enhancement of apoptosis. Chemical modification, such as by esterification, increases the bioavailability and enhances the therapeutic properties of EGCG compared to its natural form.

- Quercetin

**Figure 5:**
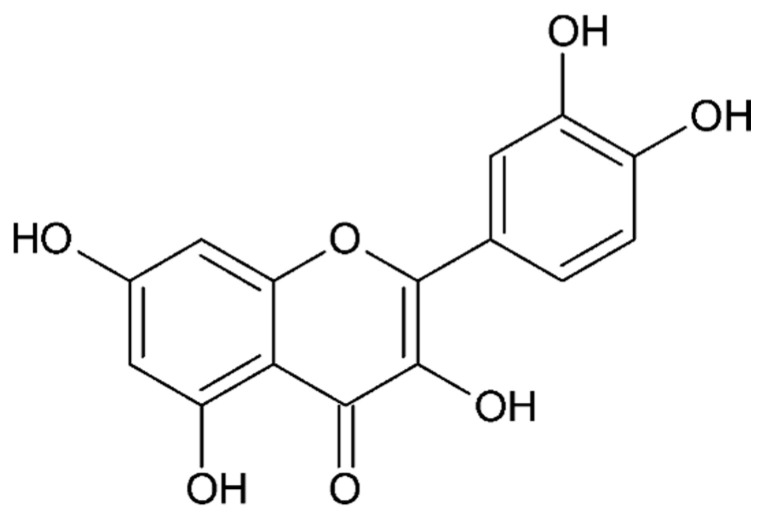
Structural formula of quercitin.

Quercetin (Figure 5), a flavonol found in fruits and vegetables (e.g., apples, berries, onions, and chili peppers), exhibits anticancer properties and anti-endometriosis activity. It inhibits cell proliferation through mechanisms such as apoptosis induction, DNA fragmentation, ROS generation, and regulation of ERK1/2, P38 MAPK, and AKT kinases [139]. Animal studies have shown that quercetin reduces the size of endometrial implants and affects the levels of FSH, LH, and estrogen hormones, which inhibit the growth of ectopic endometrium [140]. In experimental models, combining quercetin with metformin or danazol enhanced its antiproliferative effects, suggesting possible synergy, though clinical confirmation is lacking [141]. In a study on women with endometriosis, supplementation with quercetin (200 mg) in combination with curcumin and acetylcysteine for two months reduced pain and reduced the use of anti-inflammatory drugs without significant side effects.

#### 4.4.3. Flavonoids and Derivatives

In vitro and in vivo studies conducted to date have evaluated the effects of flavonoids isolated from various plants on the treatment of endometriosis [142]. Extracts from *Melilotus officinalis*, traditionally used to treat, among other things, painful menstruation and uterine swelling, containing analogs of quercetin and kemferol, reduced endometrial adhesion and levels of TNF-α, VEGF, and IL-6 in rats with experimentally induced endometriosis [143]. Similar properties were shown for flavonoids from *Urtica dioica*, known for its diuretic effects and relief of menstrual bleeding [144]. Rutin, kemferol, and their analogs from the methanolic extract of this plant reduced endometrial tissue adhesion, lesion volume, and inflammation. The optimal daily intake was estimated at 1129 mg for a person weighing 70 kg.

Flavonoids from other plants, such as Phaleria macrocarpa, Pinus pinaster (French maritime pine bark), and Pueraria lobata, also demonstrated anti-endometriotic activity in preclinical in vitro or animal models [145,146]. Of particular note is pycnogenol (pine bark extract), which, in studies on women, reduced menstrual pain from 8–9 to 2 on the VAS scale after 3 months of supplementation at a dose of 100 mg per day in combination with contraceptive therapy.

Other compounds, such as myricetin, hexahydroxyflavone, and flavonoids from Kaempferia parviflora, such as 5,7-dimethoxyflavone (DMF), were also effective [147,148]. Myricetin induced apoptosis of endometriosis cells through oxidative stress and mitochondrial dysfunction, while DMF had multiple effects, including cell cycle arrest and inactivation of the PI3K/AKT pathway.

#### 4.4.4. Ginsenoside

Ginsenosides are a group of active chemical compounds responsible for key properties of ginseng. They are natural substances categorized as steroidal glycosides and triterpene saponins, and their amount can vary depending on the species of ginseng. It is worth noting that these compounds are found only in plants of the Panax genus, which is widely distributed in Far Eastern culture, such as *Panax ginseng* C.A. Meyer, *Panax quinquefolium* L., and *Panax japonicus* C.A. Meyer. Moreover, preclinical studies suggest that ginsenosides may modulate pathways relevant to endometriosis pathophysiology, including inflammation and hormonal signaling [149,150].

Different species of Panax vary in their ginsenoside content, which affects how they can support the body. Ginsenosides exhibit numerous beneficial properties, including anti-inflammatory and antioxidant effects, suggesting their potential use in the treatment of inflammation-related diseases [150]. What is interesting, in a mouse model, administration of red ginseng altered miRNA expression, which was connected with the reduction of the size of endometrial implants [151]

#### 4.4.5. Cannabidiol

Cannabinoids are active compounds found in Cannabis sativa, a plant long used in medicine [152,153]. They are divided into three main groups: endocannabinoids produced in the body, phytocannabinoids derived from Cannabis sativa, and synthetic cannabinoids. Their effects are mainly related to CB1 and CB2 receptors. CBD, a natural and non-psychoactive cannabinoid, shows affinity for both of these receptors. In 2018, it became the first cannabinoid approved by the Food and Drug Administration (FDA) in the United States for the treatment of two types of epileptic seizures under the name Epidiolex.

**Figure 6:**
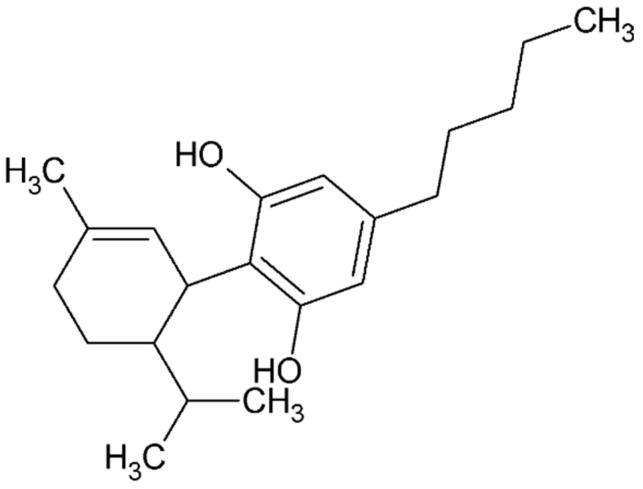
Structural formula of cannabidiol.

Studies have shown that CBD (Figure 6) inhibits endothelial cell proliferation and migration in vitro, which may impact angiogenesis relevant to endometriosis [154]. These processes play a key role in angiogenesis. This effect is related to CBD’s action against the formation of tubular structures by endothelial cells, which is important for angiogenesis. Consequently, inhibition of angiogenesis in vivo was observed in the presence of a potent angiogenic cocktail containing VEGF, TNF-α, and heparin [154]. What is more, CBD treatment significantly decreased cyst diameter, volume, and surface area. Moreover, it altered the morphology of the lesions by reducing the presence of epithelial glands and stromal tissue [155].

CBD also reduces the expression of various pro-angiogenic factors such as MMP-2, MMP-9, TIMP-1, CXCL16, endothelin-1, IL-8, and PDGF-AA [156]. In addition, cannabidiol hydroxyquinone (HU-331) has shown the ability to inhibit angiogenesis in an ex vivo model of VEGF-induced rat aortic ring. In mouse models of tumor angiogenesis, HU-331 reduced total vessel area, density, and size.

In addition, studies on human endothelial cells showed that CBD reduced the expression and levels of ICAM-1 and VCAM-1 induced by high glucose concentrations and reduced the ability of monocytes to adhere to endothelial cells. It was also found that CBD can reduce NF-κB levels. Both inflammation and angiogenesis play important roles in the pathogenesis of endometriosis, and CBD can inhibit these processes through various molecular pathways [157].

#### 4.4.6. Rosmarinic Acid

**Figure 7:**
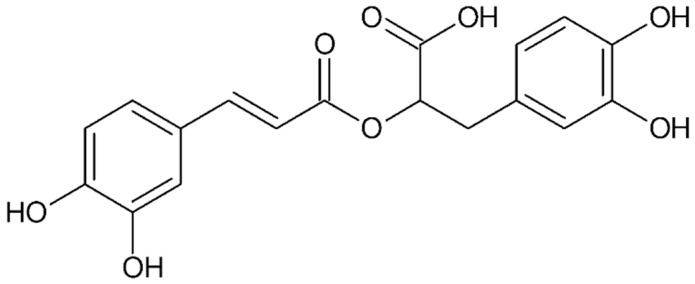
Structural formula of rosmarinic acid.

Rosmarinic acid (Figure 7), present in several plants such as rosemary (*Rosmarinus officinalis*), sage (*Salvia officinalis*), thyme (*Thymus* sp.), lemon balm (*Melissa officinalis*), basil (*Ocimum* sp.), oregano (*Origanum vulgare*), and perilla (*Perilla frutescens*, also known as Chinese basil, is a phenolic compound with a broad spectrum of health-promoting and therapeutic properties [158]. With its antioxidant, anti-inflammatory, anti-tumor, and anti-angiogenic activities, it may play an important role in the treatment of endometriosis [159]. Rosmarinic acid (RA) is an ester of caffeic acid (CA) and 3-(3,4-dihydroxyphenyl) lactic acid (DHPLA). After oral administration, it undergoes extensive pre-systemic metabolism so that only a small amount of unchanged RA enters the plasma [160,161]. In the body, it is hydrolyzed by colon bacteria into its constituent acids, CA and DHPLA.

Research by Ferelli’s team (2018) evaluated the anti-endometriosis potential of rosemary acid and carnosic acid [162]. The experiment used a culture of human endometrial lining cells and a BALB/c mouse model with induced endometriosis-like lesions. The results showed that both compounds inhibited cell proliferation and reduced the size of endometriotic lesions in mice when used at a dose of 1–3 mg/kg/day. In addition, rosmarinic acid induced apoptosis in endometrial tissue and reduced intracellular accumulation of reactive oxygen species (ROS) in human endometrial lining cells.

#### 4.4.7. Capsaicin

**Figure 8:**
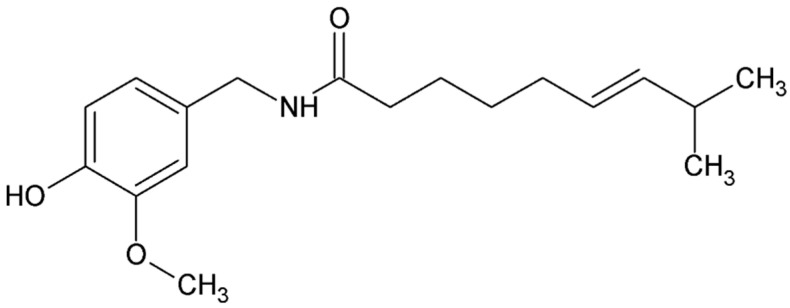
Structural formula of capsaicin.

Capsaicin (trans-8-methyl-N-vanillyl-6-nonenamide, Figure 8) is the active ingredient in chili peppers and other plants of the bell pepper family. The capsaicin content of these plants ranges from 0.1% to 2.0%, and the peppers themselves are commonly used as food additives. Topical application of capsaicin produces a burning sensation and is used as a tool to induce standardized pain in experimental studies on humans and laboratory animals.

The pharmacological effects of capsaicin, both topically and systemically administered, have been extensively studied in a variety of species [163,164]. Capsaicin has become a reliable model for evaluating the efficacy of new analgesics [165,166].

Despite its potent pain-inducing effects, this substance was used as an analgesic as early as 7000 BC [167,168]. The first formal reports of its analgesic properties date back to 1850, when an alcoholic extract of hot peppers was used to treat toothache [164,169]. Through years of research, capsaicin has been recognized as an effective, non-opioid analgesic with few side effects. In addition to culinary uses, capsaicin has found therapeutic applications in the treatment of various conditions, such as pruritus in psoriasis, obesity, urological diseases, respiratory, cardiovascular, gastrointestinal ailments, and cancer [170,171]. In vitro findings show that capsaicin selectively inhibits proliferation of endometriotic cells more effectively than normal endometrial cells, suggesting potential therapeutic relevance, although clinical data are currently lacking [172].

#### 4.4.8. Vitamins

**Figure 9:**
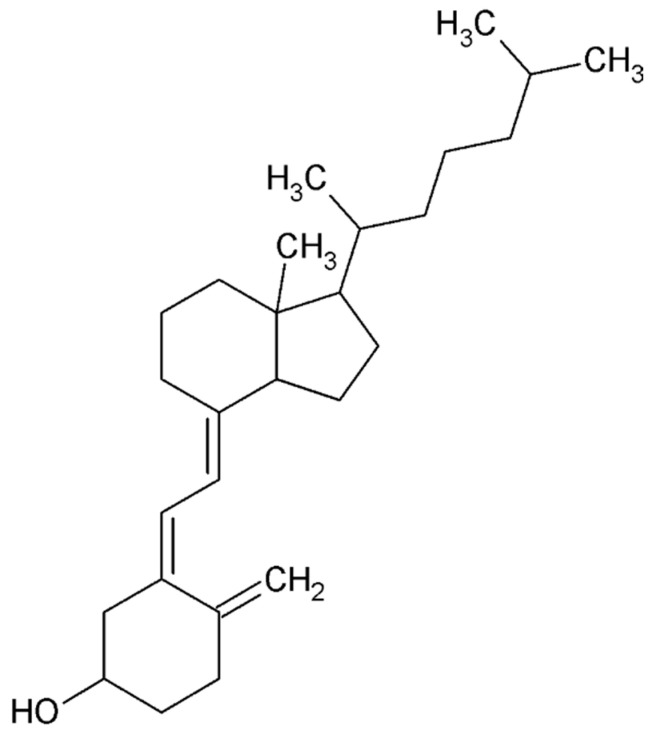
Structural formula of vitamin D.

Vitamin D (Figure 9) plays a key role in regulating blood calcium levels and modulating the immune system. Its main sources are oily fish, fish oils, and supplements, but the most effective way to obtain vitamin D is through its dermal synthesis under UVB radiation. Since endometriosis is a chronic inflammatory disease resulting from immune system dysfunction, investigating the effect of vitamin D as an immune modulator on the development of this disease seems a promising direction, especially in the context of its association with autoimmune and inflammatory diseases.

A 2020 meta-analysis found that low levels of vitamin D may be associated with an increased risk of developing endometriosis and exacerbating its symptoms [173]. Similar correlations have been confirmed in other studies, including between vitamin D levels in women of childbearing age and the size of endometrial ovarian lesions [2,3,4,5,174,175,176,177,178]. However, some studies have found no such correlations [178,179].

**Figure 10:**
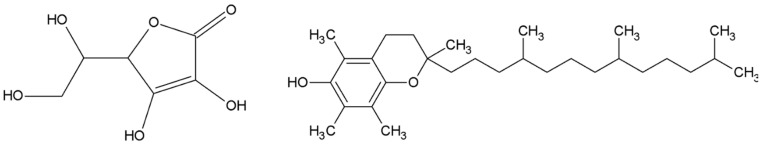
Structural formulas of vitamin C and vitamin E.

Vitamins C and E (Figure 10) play an important role in neutralizing free radicals through their antioxidant properties. Vitamin C, being an exogenous component, must be supplied through diet or supplementation. Its rich sources are rosehips, black currants, strawberries, citrus fruits, parsley, and peppers. Fat-soluble vitamin E is found in nuts, seeds, vegetable oils, green leafy vegetables, and cereals [180].

Hoorsan et al. demonstrated in a mouse study that vitamin C enhances ovarian function and suppresses the induction and growth of endometrial grafts [181]. Similar findings were observed in rats, where intravenous vitamin C inhibited the formation of endometriotic implants and reduced their volume [182]. Additionally, research on the effects of vitamins C and E on VEGF gene expression and production in peritoneal macrophages of women with endometriosis revealed that these vitamins could alter VEGF gene expression at varying incubation times and concentrations but did not influence VEGF production [183].

It also confirmed the beneficial effects of vitamins C and E in reducing endometriosis symptoms compared to placebo when used at a dose of 1000 mg/day and 800 UI/day, respectively [184,185]. Furthermore, a prospective cohort study of 1383 cases of laparoscopically confirmed endometriosis, indicating an inverse relationship between dietary intake of vitamins C, E, thiamine, and folic acid, as well as the risk of endometriosis. However, the authors suggested that this protective effect may not be directly attributed to the vitamins themselves but rather to other essential nutrients present in the diet [186].

While a wide range of natural compounds have demonstrated anti-inflammatory, antioxidant, and antiangiogenic effects relevant to endometriosis pathogenesis, most supporting data to date are derived from preclinical studies. Clinical trials are still needed to confirm their efficacy and safety in human populations and determine optimal formulations, doses, and combinations with existing treatments.

### 4.5. Small Compounds Used in the Treatment

Endometriosis is a challenging condition to manage, often requiring a combination of medical, surgical, and alternative approaches.

Hormonal therapies targeting the estrogen and progesterone axis—such as GnRH analogs, progestins, and oral contraceptives—remain among the most widely used and effective pharmacological options for managing endometriosis symptoms and slowing disease progression. However, it is important to note that despite their therapeutic potential, these treatments are generally contraindicated in patients seeking to preserve or improve fertility. By suppressing ovulation and modulating endometrial receptivity, they may interfere with reproductive goals and, thus, require careful consideration in women of reproductive age who wish to conceive. Therefore, any hormonal intervention should be evaluated not only in terms of symptom control but also within the broader context of the patient’s reproductive intentions.

Experimental treatments, aiming to improve efficacy and reduce side effects compared to current standard therapies, are continually being explored. Here are some of the promising experimental treatment methods and targets (Table 4):

#### 4.5.1. Selective Progesterone Receptor Modulators (SPRMs)

Selective Progesterone Receptor Modulators (SPRMs) are a class of drugs that act on progesterone receptors with both agonist and antagonist effects. Ulipristal acetate, a well-known SPRM, has shown potential in reducing endometrial tissue proliferation and associated pain [187].

Mifepristone (DRUGBANK: DB00834) was the first selective progesterone receptor modulator, discovered in the 1980s by the French pharmaceutical company Roussel-Uclaf during research on anti-glucocorticoid drugs. Synthesized by Georges Teutsch, it was originally designated as RU-38486, later shortened to RU-486. Its antiprogestin properties were investigated by endocrinologist Étienne-Émile Baulieu, who played a key role in its development as a medication for pregnancy termination, earning him the title of the “father” of the abortion pill. Mifepristone is a synthetic estrane steroid, chemically named 11β-(4-(dimethylamino) phenyl)-17α-(1-propynyl) estra-4,9-dien-17β-ol-3-one [188].

UPA Ulipristal acetate (DRUGBANK: DB08867) is a steroidal selective progesterone receptor modulator (SPRM) that was initially studied in the 1990s as an antifertility drug, similar to mifepristone. Originally developed by the National Institute of Child Health and Human Development (NICHD), UPA is derived from 19-norprogesterone. Research in animal models has shown that UPA may induce regression of endometriotic lesions, reduce cell proliferation, and decrease inflammation. Clinical reports suggest its potential in alleviating chronic pelvic pain associated with endometriosis, although its effects on different subtypes of the condition remain uncertain. Due to conflicting evidence regarding its efficacy, further research through well-designed randomized controlled trials (RCTs) is necessary [188].

Vilaprisan (DRUGBANK: DB11971) is a potent, orally active selective progesterone receptor modulator (SPRM) developed by Bayer AG. Structurally, it is a 17-hydroxy-17-pentafluoroethyl-estra-4,9(10)-dien-11-aryl derivative, with weak binding to the glucocorticoid and androgen receptors but no effect on the estrogen receptor. Vilaprisan has been investigated in clinical trials for the treatment of heavy menstrual bleeding (HMB) associated with uterine fibroids and for managing symptomatic endometriosis. However, all clinical trials were put on hold due to safety concerns identified in long-term toxicology studies in rodents [188].

#### 4.5.2. Estrogen Receptors

Since endometriosis is a hormone-dependent condition, estrogen receptors play a pivotal role in the pathogenesis and progression of endometriosis by mediating the effect of estrogen [104].

Previous studies conducted on mouse models and endometrioma cells from patients have elucidated the molecular mechanisms underlying the specific roles of each estrogen receptor (ER) isoform in the initiation and progression of the disease. Beliard et al. observed a positive correlation between cell proliferation and ER levels in normal and eutopic endometrium obtained from patients aged 26 to 40 years, although they found no correlation with apoptosis and estrogen receptor levels [196]. Notably, the study did not specify the antibody used, which limited the differentiation between ERα and ERβ. Consequently, researchers utilized ERα and ERβ knockout mice to induce endometriosis-like lesions by injecting minced uterine tissue into the peritoneal cavity of syngeneic host mice [189]. These experiments demonstrated that both ER isoforms are essential for the growth of endometriotic-like lesions. However, estradiol significantly enhanced the development of these lesions, predominantly highlighting the necessity of ERα for cell adhesion, proliferation, and neoangiogenesis that support lesion growth. The impact of ERβ knockout on ectopic lesion growth was less significant compared to the effects of ERα knockout.

Bazedoxifene (DRUGBANK: DB06401). The selective estrogen receptor modulator (SERM) bazedoxifene (BZA) effectively inhibits estrogen-induced stimulation of the uterine endometrium while preserving estrogenic effects in bone and the central nervous system [190]. These characteristics make it a promising candidate for treating endometriosis. In an experimental model, endometriosis was induced in reproductive-age CD-1 mice. After treatment, the size of the endometriotic implants was measured, revealing a mean size of 60 mm^2^ in the control group compared to 21 mm^2^ in the BZA treatment group.

SR-16234 (DRUGBANK: DB05966). Treatment with SR significantly decreased both the total number and size of lesions per mouse without promoting endometrial growth [191]. Additionally, SR downregulated the mRNA expression of LPS-enhanced Vegf, Il-6, Ptgs-2, Ccl-2, and ER in endometriosis-like lesions. Immunohistochemical analysis revealed a reduction in the percentage of Ki67-positive cells, as well as a decrease in the intensity and proportion of cells positively expressing ERα, CD3, F4/80, and PECAM following SR treatment. Furthermore, SR treatment led to a reduction in the expression levels of NF-κB p65 and phosphorylated NF-κB p65.

The compound was also tested in a clinical trial. After 12 weeks of oral administration of SR-16234 at a dosage of 40 mg, there were statistically significant reductions in pelvic pain measured by the Visual Analog Scale (VAS), total pelvic pain score, total dysmenorrhea score, stiffness of Douglas’ pouch, and limitations in uterine movement compared to baseline values [192]. This trial indicates that a selective estrogen receptor modulator may be an effective treatment for pain associated with endometriosis.

#### 4.5.3. Steroid Sulfatase Inhibitors

Steroid sulfatase (STS) is an enzyme that plays a crucial role in the local regulation of estrogen levels by hydrolyzing sulfate conjugates of steroids, such as estrone sulfate, into their active forms, like estrone. This enzymatic activity is particularly relevant in the context of estrogen-dependent conditions, including endometriosis.

The steroid sulfatase pathway provides an alternative source of local estrogens within endometriotic lesions, promoting cell proliferation, inflammation, and the associated symptoms of endometriosis, such as pain and infertility. By converting estrone sulfate to estrone, which can subsequently be converted to the more potent estrogen, estradiol, STS contributes to the local estrogen pool. This local production of estrogen fuels the growth and persistence of endometriotic tissue [193,194].

Moreover, the local estrogen production contributes to a pro-inflammatory environment by inducing the production of cytokines, chemokines, and prostaglandins. These inflammatory mediators not only promote the survival and growth of endometriotic lesions but also contribute to pain and other symptoms associated with the condition [195].

Steroid sulfatase inhibitors, as well as combining steroid sulfatase inhibitors with other hormonal therapies, such as aromatase inhibitors or GnRH analogs, may offer synergistic effects in reducing estrogen levels and managing endometriosis symptoms [197]. This multi-targeted approach could enhance treatment efficacy and provide relief for patients who do not respond adequately to conventional therapies.

STS inhibitors include steroidal and non-steroidal compounds. Among the non-steroidal STS inhibitors, the most important role is played by the group of Coumarin Sulfamate derivatives, which includes molecules possessing a fused tricyclic structure; Irosustat (STX64) is considered the most potent [198,199]. It is the first non-steroidal STS inhibitor that entered the clinical phase in postmenopausal women with breast cancer.

Among steroidal inhibitors, estrone-3-O-sulfamate (EMATE, MCE: 148672-09-7) and its derivatives are considered one of the most potent. E2MATE (estradiol-3-O-sulfamate, MCE: 172377-52-5 https://www.medchemexpress.com/cas/172377-52-5.html (accessed on 16 May 2025)) efficiently suppresses STS activity in endometrial tissue both in vitro and in vivo [199].

#### 4.5.4. Aromatase Inhibitors

Aromatase inhibitors, typically used in breast cancer treatment, inhibit the enzyme aromatase, which converts androgens to estrogens. This reduction in local estrogen production can potentially decrease endometriotic tissue growth and alleviate symptoms [200]. Here are some III-generation inhibitors of aromatase commonly used.

Letrozole (Femara, Drugbank: DB01006)—A third-generation aromatase inhibitor, it is presented as effective in reducing pain associated with endometriosis, often used in combination with other therapies (gestagens, oral contraceptives, or GnRH agonists) [201]. A meta-analysis showed its effectiveness in decreasing pain and improving quality of life.

Anastrozole (Arimidex, Drugbank: DB01217)—This drug is presented as effective in reducing pain associated with endometriosis [202]. A meta-analysis cited in the article demonstrated its effectiveness in decreasing pain, reducing lesion size, and improving quality of life, often used in combination with other treatments. A specific study highlighted its use with goserelin (a GnRH agonist), demonstrating a significant reduction in pain compared to goserelin alone. It was found to reduce pain in postmenopausal patients with endometriosis, but it failed to treat a case of ureteral endometriosis due to significant fibrosis. A side effect is that bone mineral density decreased in one patient after 9 months of treatment.

Because of the fact that these medications are usually recommended for women who are not seeking to conceive, as they can interfere with fertility, treatment should be guided by a healthcare professional who can consider individual circumstances and other treatment options, such as hormonal therapies or surgical management [197].

#### 4.5.5. GnRH Antagonists

Gonadotropin-releasing hormone (GnRH) antagonists directly inhibit GnRH receptors, reducing the release of follicle-stimulating hormone (FSH) and luteinizing hormone (LH), which, in turn, lowers estrogen levels.

Elagolix (Orilissa, Drugbank: DB11979)—This is a GnRH antagonist specifically approved for the management of moderate to severe endometriosis-related pain [203]. It works by reducing the release of hormones that promote ovarian function and estrogen production.

Degarelix (Firmagon, Drugbank: DB06699)—While primarily used in the treatment of prostate cancer, it is a GnRH antagonist and has been studied for potential use in endometriosis, though it is not commonly used for this indication [204].

Buserelin (Suprefact, Drugbank: DB06719)—Although more often classified as a GnRH agonist, it can have antagonistic effects when used at specific doses [205]. It may be employed off-label in endometriosis treatment settings.

While GnRH antagonists can be effective in treating endometriosis, they can cause side effects related to estrogen deficiency, such as bone density loss. Treatment should be managed by a healthcare professional, taking individual patient needs and potential side effects into account.

#### 4.5.6. Anti-Angiogenic Agents

Anti-angiogenic agents are being explored as potential treatments for endometriosis due to their ability to inhibit the formation of new blood vessels, which is a crucial aspect of endometrial tissue growth [206]. Some anti-angiogenic agents that have been studied in the context of endometriosis include:

Bevacizumab—Bevacizumab (Drugbank: DB00112) is a monoclonal antibody that inhibits vascular endothelial growth factor (VEGF). Some studies have suggested that it may be effective in reducing endometriosis-related pain and improving quality of life [207].

Thalidomide—The current findings, along with data from a few published studies, highlight the potential of thalidomide (Drugbank: DB01041) as a treatment for endometriosis [208]. This supports the hypothesis that thalidomide can cause regression in the progression of endometriosis, emphasizing the need for further research to proceed cautiously due to its teratogenic history. Thalidomide effectively reduced the lesion area and the CPI of peritoneal endometriotic implants in rats at both 1 mg/kg/day and 10 mg/kg/day doses, with the lower dose proving as effective as the higher, teratogenic dose. Consequently, thalidomide should be considered a potential treatment option for endometriosis in women.

Sunitinib—Sunitinib (Drugbank: DB01268) was found to inhibit endometriotic lesions by promoting the maturation of myeloid-derived suppressor cells (MDSCs) in peritoneal fluid and inhibiting their immunosuppressive functions [209]. By altering the immune microenvironment, sunitinib hindered the progression of endometriosis. This suggests that sunitinib could have potential therapeutic effects as a novel immunotherapy by promoting the maturation, differentiation, and metabolism of MDSCs for treating endometriosis.

#### 4.5.7. Immune Modulators

Immune modulators target the immune dysregulation associated with endometriosis. Pentoxifylline, which inhibits the production of inflammatory cytokines, has been studied for its potential to reduce endometriosis-related symptoms.

Danazol—Danazol (Drugbank: DB01406) is a treatment option for endometriosis, working by creating a high androgen/low estrogen environment akin to pseudo menopause [210]. This hormonal shift leads to the atrophy of endometriotic implants, resulting in the alleviation of painful symptoms. Studies have shown that danazol, even when used alongside surgical therapy, effectively reduces endometriosis-related pain compared to a placebo. It also improves laparoscopic scores more significantly than placebo or no treatment. However, patients using danazol report side effects more frequently than those on placebo, primarily due to its androgenic effects, which limit its use despite its effectiveness in managing the symptoms and signs of endometriosis.

PD-1/PDL-1 (Programmed cell death protein 1/death ligand 1) inhibitors—Endometriosis stimulates bone marrow mesenchymal stem cell differentiation via paracrine signal transduction and increases PD-1 expression in T cells [211]. Increased PD-1 expression may be a mechanism by which endometrial tissue evades immune monitoring. Targeted PD-1 inhibition may be an effective treatment for endometriosis [212].

CD47 inhibitors—Overexpression of CD47 molecules on the surface of endometriosis cells is associated with their recognition by macrophages as normal cells [213]. Blocking this signal with the use of siRNA or neutralizing antibodies may serve to switch macrophages to phagocytosis of endometriosis cells.

Azathioprine—The use of this immunomodulator (Drugbank: DB00993) could also be considered, as the findings show that immunosuppression for 3 months accelerated the progression of spontaneous endometriosis in the baboon model but did not influence the development of induced endometriosis, nor did it induce the disease in baboons with a previously confirmed normal pelvis [214].

### 4.6. Stem Cell Therapy

Stem cell therapy is an emerging field in endometriosis treatment. Mesenchymal stem cells (MSCs) have shown promise in preclinical studies for their potential to modulate immune responses and repair tissue damage associated with endometriosis [215].

Stem cells (SCs) play a significant role in the development and progression of endometriosis by being recruited to ectopic foci from retrograde menstrual fluid and bone marrow. Targeting SC recruitment has been identified as a promising approach to inhibit the progression of this disease. Bazedoxifene, a selective modulator of estrogen receptors, has demonstrated the ability to inhibit ectopic endometrial growth and interfere with SC recruitment in mice. In experimental studies, the combination of bazedoxifene with conjugated estrogens significantly reduced SC recruitment to lesions, leading to a notable reduction in lesion size while sparing SC recruitment to the uterus in control animals [190]. Additionally, endothelial progenitor cells (EPCs) derived from bone marrow contribute to neo-vasculogenesis in endometriotic lesions, with their recruitment being mediated by the CXCR4/SDF1 signaling axis under hypoxic conditions [216]. Blocking CXCR4 with AMD 3100 effectively reduces EPC recruitment and decreases capillary density within the lesions. Estradiol has also been shown to stimulate the release of CXCL12 and CXCR4, promoting SC migration, a process that can be inhibited by AMD 3100. Conventional progestin treatments may further support this strategy by suppressing CXCL12 production, thereby limiting SC recruitment to ectopic foci. These findings suggest that inhibiting SC recruitment, through safe and effective interventions, represents a relevant therapeutic strategy in endometriosis management [21,217,218].

### 4.7. Gene Therapy

Gene therapy represents a promising approach for the treatment of endometriosis, utilizing advanced techniques such as nanotechnology, microRNAs, and other molecular strategies. Numerous studies to date have demonstrated the potential of nanotechnology-based gene therapy in managing this condition. One notable example involves the application of nanocarriers in gene delivery systems for endometriosis, including polymeric nanoparticles, cell-penetrating peptides (CPPs), and extracellular vesicles (EVs). These nanocarriers facilitate the targeted delivery of therapeutic genes or siRNA to endometrial lesions [219]. Othman et al. employed an adenoviral vector to deliver the dominant negative estrogen receptor (DN-ER) gene into human endometriotic cells in vitro. This intervention led to the suppression of cell proliferation, reduced cytokine production, and the induction of apoptosis as a consequence of disrupted estrogen signaling [220]. Another approach includes the use of siRNA carriers. For example, Egorova et al. showed inhibition of vascularization in endometrial lesions after the use of CXCR4 receptor-targeted siRNA carrier L1 [221]. Techniques such as CRISPR-Cas9 are also being investigated for their ability to target specific genetic abnormalities in endometriotic cells [222]. They could be used for so-called gene editing. Some genes, for example, the gene-encoding neuropeptide S receptor 1, are promising targets. Tapmeier et al. observed a correlation between the deletion at rs142885915 in this gene and the occurrence of Stages III and IV of endometriosis. Moreover, the inhibition of NPSR1 through a selective inhibitor, SHA 68R, reduced immune response in mice [223].

### 4.8. Clinical Trials

Clinical trials targeting new therapies for endometriosis are of significant importance. Today, they are a driving force in discovering new drugs and therapeutic approaches. Below, we present clinical trials conducted from 2024 to the present, focused on investigating new substances and/or their combinations (Table 5). The primary goal of these studies is to develop therapies that control pain and those aimed at treating the disease itself.

#### 4.8.1. Relugolix Combination Therapy

Several studies examine relugolix combination therapy (relugolix 40 mg, estradiol 1 mg, and norethindrone acetate 0.5 mg) [228,229,230]. The long-term extension of the SPIRIT trials (Becker et al.) showed sustained improvements in endometriosis-associated pain, dyspareunia, and function over 2 years. Adams ED summarizes the SPIRIT 1 and 2 trials, which were replicate phase 3 studies examining relugolix combination therapy versus placebo in patients with endometriosis-associated pain. As-Sanie et al. also demonstrated the impact of relugolix combination therapy on functioning and quality of life in women with endometriosis-associated pain.

#### 4.8.2. Linzagolix

The EDELWEISS 3 trial evaluated linzagolix at 75 mg alone or 200 mg in combination with add-back therapy (ABT). The study found that 200 mg linzagolix with ABT significantly reduced dysmenorrhea and non-menstrual pelvic pain at 3 months, while 75 mg linzagolix significantly decreased dysmenorrhea only [231].

#### 4.8.3. Gefapixant

A randomized trial assessed gefapixant (45 mg twice daily), a P2X3 receptor antagonist, for treating endometriosis-related pain. The study was inconclusive, with results directionally favoring gefapixant but not demonstrating superiority to placebo [224].

#### 4.8.4. Eliapixant

The SCHUMANN study evaluated eliapixant, another P2X3 antagonist, for endometriosis-associated pelvic pain. The study was terminated early and did not meet its primary objective, showing no significant differences in pain reduction compared to placebo [225].

#### 4.8.5. Anakinra

A pilot study evaluated anakinra, an interleukin-1 (IL-1) antagonist, and found trends toward improvement in quality of life and dysmenorrhea, justifying further investigation [226].

#### 4.8.6. Quinagolide

The QLARITY trial assessed a quinagolide vaginal ring but found no significant difference in reducing total lesion size compared to placebo [227].

#### 4.8.7. Norethindrone Acetate (NETA) vs. Dienogest

A study by Gurbuz et al. compared NETA and dienogest for pain relief. Both progestins effectively reduced pain, but NETA achieved a greater reduction in endometrioma size and had a lower dropout rate [115].

#### 4.8.8. 99mTc-Maraciclatide

Ongoing clinical trial with ID NCT05623332 (ClinicalTrials.gov), University of Oxford. Also, 99mTc-maraciclatide is a radiolabeled tracer currently under investigation as a non-invasive diagnostic tool for endometriosis, especially superficial peritoneal endometriosis. It binds to αvβ3 integrin, a protein involved in angiogenesis, which is essential for the growth of endometriotic lesions. Early studies indicate that it can effectively visualize these lesions and correlate well with surgical and histological results, potentially shortening the delay in diagnosing endometriosis.

## 5. Impact on Quality of Life

Endometriosis can significantly impact a woman’s quality of life, affecting her physical, emotional, and social well-being. Chronic pain and fatigue can limit daily activities and productivity, leading to absenteeism from work or school. The emotional burden of dealing with a chronic, often misunderstood condition can result in anxiety, depression, and strained relationships [65]. Therefore, a multidisciplinary approach involving pain specialists, gynecologists, and mental health professionals is often beneficial in managing the comprehensive effects of the disease.

## 6. Conclusions

Endometriosis is a complex, multifactorial disorder. The pathogenetic mechanisms of this disease include retrograde menstruation, spread through the lymphatic and circulatory systems, the stem cell theory, and genetic and epigenetic factors. The diagnosis of endometriosis continues to be a challenge due to the lack of correlation between the severity of symptoms and the extent of the disease, with over 25% of women remaining asymptomatic. Identification of protein or microRNA biomarkers, as well as correlations between them, could significantly improve early diagnosis and treatment personalization.

Current therapeutic methods include pharmacological, surgical, and alternative approaches, with the best results achieved through a combination of these methods depending on the nature of the individual case. New therapeutic directions, including selective progesterone receptor modulators, aromatase inhibitors, GnRH antagonists, and gene therapy, are particularly promising.

Endometriosis significantly impacts patients’ quality of life, causing chronic pain, mental health disorders, and social problems, which require a multidisciplinary therapeutic approach involving pain specialists, gynecologists, and mental health professionals.

## 7. Future Perspective

The future of endometriosis treatment rests on three main pillars: advanced molecular diagnostics utilizing biomarker panels and artificial intelligence for the analysis of protein profiles, innovative pharmacological strategies, including selective progesterone receptor modulators (SPRMs) and next-generation GnRH antagonists, and targeted therapies such as angiogenesis inhibitors and modulators of the immune system.

Regenerative medicine using stem cells and gene therapy opens up opportunities for precise treatment, while personalized medicine with machine-learning algorithms will enable the selection of optimal therapy based on the patient’s individual genetic profile.

The future is a multidisciplinary approach combining advanced diagnostic technologies, innovative targeted therapies, and holistic care, which can significantly improve prognosis, treatment, and quality of life for women with endometriosis.

## 8. Search Methodology

This review is based on scientific publications in English available in the major biomedical databases, including PubMed, ScienceDirect, and Wiley Library. These databases were searched using relevant keywords such as endometriosis, biomarkers (and specific names), treatment, natural substances (and specific names), inhibitor, molecular target (by name), surgery, pain management, psychology, and endometriosis, and mechanisms of pathogenesis, all in various combinations. The selection of publications was conducted in two stages. First, titles and abstracts were reviewed. In the second stage, full texts were analyzed. Studies deemed unrelated to the review’s focus were excluded.

## Figures and Tables

**Table 1:** Protein and non-protein biomarkers of endometriosis.

Biomarker	Uniprot Number	Description	Reference(s)
CA 125	P04633	A tumor-associated antigen overexpressed in ovarian cancer and endometriosis	[62,63,64]
Glycodelin	P04633	A glycoprotein secreted by the testis that regulates sperm motility and capacitation	[69,70,71,72,73,74]
HE4	P04633	A secreted glycoprotein overexpressed in serous and endometrioid ovarian cancer	[75,76]
IL-6	P04633	A cytokine involved in inflammation and immune response	[77,78]
VCAM-1	P04633	A transmembrane glycoprotein involved in immune cell adhesion and recruitment	[79,80,81,82,83]
VEGF	P04633	A growth factor involved in angiogenesis and endometriosis tissue growth	[84]
MCP-1	P04633	A chemokine involved in immune cell recruitment and inflammation	[85,86]
Urocortin	P04633	A neuropeptide involved in stress response and inflammation	[87]
CA 19-9	P04633	A tumor-associated antigen is overexpressed in some types of cancer and endometriosis	[88,89]
Estrogen level	N/A	The level of estrogen in the body, which is increased in response to endometriosis	[90]

**Table 2:** Examples of pharmacotherapy for endometriosis.

Pharmacological Treatments			
Treatment	Route of Administration	Advantages	Disadvantages
NSAIDs (e.g., ibuprofen, naproxen)	Oral	Easily accessible; reduces inflammation and dysmenorrhea	Symptom-only relief; no effect on lesion growth; GI side effects
Combined Oral Contraceptives (COCs)	Oral, Vaginal ring, Transdermal patch	First-line therapy; regulates cycles; reduces pain and bleeding	Limited in severe disease; risk of VTE; may not prevent progression
Progestins (Dienogest, NETA, MPA)	Oral, Injectable, Intrauterine	Inhibits endometrial growth; suitable for long-term use; affordable	Breakthrough bleeding; weight gain; ↓ BMD; some forms carry VTE and liver risks
GnRH Agonists (leuprorelin, nafarelin)	Injection, Nasal spray	Highly effective pain relief; suppresses estrogen	Hypoestrogenism (hot flashes, osteopenia); requires add-back therapy; not suitable for conception
GnRH Antagonists (elagolix, relugolix)	Oral	Rapid onset; avoids flare effect of agonists	Similar side effects as agonists; limited long-term safety data
Danazol/Gestrinone	Oral	Induces pseudomenopause; halts lesion growth	Androgenic effects (weight gain, acne, voice change); liver toxicity
Aromatase Inhibitors (letrozole, anastrozole)	Oral	Suppresses local estrogen; used in refractory cases	Vaginal dryness, osteoporosis risk; usually combined with other agents
LNG-IUS (Levonorgestrel IUD)	Intrauterine	Local hormone delivery; reduces pelvic pain; low systemic exposure	Limited effect on extrauterine lesions; irregular bleeding; insertion discomfort
Resveratrol (experimental)	Oral (natural compound)	Potential anti-inflammatory and apoptotic effects	Limited clinical evidence; currently investigational

↓—decrease.

**Table 3:** Examples of surgical treatment of endometriosis.

Surgical Procedure	Route/Approach	Advantages	Disadvantages
Laparoscopic Excision/Ablation	Minimally invasive surgery	Gold standard for diagnosis and treatment; removes lesions; symptom relief	Requires expertise; recurrence possible; operative risks
Ovarian Cystectomy (Endometrioma)	Laparoscopic surgery	Lower recurrence vs. drainage; preserves fertility	↓ AMH; technical difficulty; risk of ovarian damage
Laser Vaporization	Laparoscopic (precision laser)	Minimal thermal damage; useful for superficial lesions	May not treat deep lesions; specialized equipment needed
Deep Infiltrating Endometriosis Resection	Laparoscopy or laparotomy	Improves pain and function in DIE; multidisciplinary approach recommended	Risk of complications (bowel, bladder); complex and time-consuming
Hysterectomy ± Oophorectomy	Laparoscopic, vaginal, or abdominal surgery	Option for severe pain in women done with childbearing	Permanent infertility; possible symptom persistence if extrauterine disease remains
Sclerotherapy (Endometrioma)	Ultrasound-guided aspiration + alcohol	Minimally invasive; preserves ovarian tissue	High recurrence; not standard of care; best for selected cases only
Postoperative Hormonal Suppression	Oral or IUD (COCs, progestins, GnRH analogs)	Prevents recurrence after surgery; improves long-term outcomes	Requires long-term compliance; not suitable for conception

↓—decrease.

**Table 4:** A list of small molecules used in the treatment of endometriosis.

Name	DRUGBANK Number	Short Description	Reference(s)
Mifepristone	DB00834	Synthetic estrane steroid, originally designed as an anti-glucocorticoid drug	[171]
UPA	DB08867	Steroidal selective progesterone receptor modulator (SPRM) was initially studied as an antifertility drug	[171]
Vilaprisan	DB11971	Potent, orally active selective progesterone receptor modulator (SPRM) developed by Bayer AG	[171]
Bazedoxifene	DB06401	Selective estrogen receptor modulator (SERM) that inhibits estrogen-induced stimulation of the uterine endometrium	[174]
SR-16234	DB05966	Treatment with SR significantly decreased both the total number and size of lesions per mouse without promoting endometrial growth	[175]
Irosustat	N/A	Steroid sulfatase inhibitor	[181,182]
Estrone-3-O-sulfamate	N/A	Steroid sulfatase inhibitor	[182]
E2MATE	N/A	Steroid sulfatase inhibitor	[182]
Letrozole	N/A	Aromatase inhibitor	[184]
Anastrozole	N/A	Aromatase inhibitor	[185]
Elagolix	N/A	GnRH antagonist	[186]
Degarelix	N/A	GnRH antagonist	[187]
Buserelin	N/A	GnRH antagonist	[188]
Bevacizumab	N/A	Anti-angiogenic agent	[189]
Thalidomide	N/A	Anti-angiogenic agent	[190]
Sunitinib	N/A	Anti-angiogenic agent	[191]
Danazol	N/A	Immune modulator	[192]
PD-1/PDL-1	N/A	Immune modulator	[193]
CD47 inhibitors	N/A	Immune modulator	[194]
Azathioprine	N/A	Immune modulator	[195]

**Table 5:** Recent clinical trials on endometriosis.

Substance	Study	Final Outcomes
Gefapixant	[224]	Inconclusive, results directionally favoring gefapixant but not demonstrating superiority to placebo
Eliapixant	[225]	Terminated early, no significant differences in pain reduction compared to placebo
Anakinra	[226]	Trends toward improvement in quality of life and dysmenorrhea, justifying further investigation
Quinagolide	[227]	No significant difference in reducing total lesion size compared to placebo
Norethindrone Acetate (NETA)	[115]	Effectively reduced pain, achieved a greater reduction in endometrioma size and had a lower dropout rate
99mTc-maraciclatide	N/A	N/A
Relugolix Combination Therapy	[228,229,230]	Sustained improvements in endometriosis-associated pain, dyspareunia, and function over 2 years
Linzagolix	[231]	Significantly reduced dysmenorrhea and non-menstrual pelvic pain at 3 months with 200 mg linzagolix with add-back therapy, significantly decreased dysmenorrhea only with 75 mg linzagolix
